# Can label or protein deuteration extend the phase relaxation time of Gd(III) spin labels?

**DOI:** 10.5194/mr-6-211-2025

**Published:** 2025-08-12

**Authors:** Elena Edinach, Xing Zhang, Chao-Yu Cui, Yin Yang, George Mitrikas, Alexey Bogdanov, Xun-Cheng Su, Daniella Goldfarb

**Affiliations:** 1 Department of Chemical and Biological Physics, Weizmann Institute of Science, 76100 Rehovot, Israel; 2 State Key Laboratory of Elemento-Organic Chemistry, College of Chemistry, Nankai University, 300071 Tianjin, China; 3 Institute of Nanoscience and Nanotechnology, NCSR Demokritos, Athens 15310, Greece

## Abstract

Pulse-dipolar electron paramagnetic resonance (PD-EPR) has emerged as an effective tool in structural biology, enabling distance measurements between spin labels attached to biomolecules. The sensitivity and accessible distance range of these measurements are governed by the phase memory time ( $T_{m}$ ) of the spin labels. Understanding the decoherence mechanisms affecting $T_{m}$ is crucial for optimizing sample preparation and spin-label design. This study investigates the phase relaxation behavior of two Gd(III) spin-label complexes, Gd-PyMTA and Gd-TPMTA, with various degrees of deuteration. These two complexes have significantly different zero-field-splitting (ZFS) parameters. Hahn echo decay and dynamical decoupling (DD) measurements were performed at W-band (95 GHz) in deuterated solvents (D_2_O / glycerol-d_8_), both for the free complexes and when conjugated to proteins. The impact of temperature, concentration, and field position within the EPR spectrum on $T_{m}$ was examined. Results indicate that protons within 5 Å of the Gd(III) ion do not contribute to nuclear spin diffusion (NSD), and protein deuteration offers minimal enhancement in $T_{m}$ . The dominant phase relaxation mechanisms identified at low concentrations were direct spin-lattice relaxation ( $T_{1}$ ) and transient ZFS (tZFS) fluctuations. Dynamical decoupling (DD) measurements, using the Carr–Purcell sequence with ∼ 140 refocusing pulses, resolved the presence of two populations: one with a long phase relaxation time, $T_{m,s}$ , and the other with a short one, $T_{m,f}$ . The dominating mechanism for the slowly relaxing population is direct- $T_{1}$ . $T_{m,s}$ showed no concentration dependence and was longer by a factor of about 2 than $T_{m}$ for both complexes. We tentatively assign the increase in $T_{m,s}$ to full suppression of the residual indirect- $T_{1}$ -induced spectral diffusion and NSD mechanisms. For the fast-relaxing population, $T_{m,f}$ is shorter for Gd-TPMTA; therefore, we assign it to populations for which the tZFS mechanism dominates. Because of the relatively short $T_{1}$ and the contribution of the tZFS mechanism, protein deuteration does not significantly affect $T_{m}$ .

## 1 Introduction

Distance measurements between two spin labels attached at specific sites in biomolecules, determined by pulse-dipolar electron paramagnetic resonance (PD-EPR) methods, have become standard tools in structural biology. The sensitivity of these measurements and the distance they can access depend on the phase memory time, $T_{m}$ , of the spin labels used. Accordingly, understanding the decoherence mechanisms is essential for optimizing sample preparation conditions (concentrations, solvent composition, and deuteration) and designing spin labels, thus reaching a $T_{m}$ that is as long as possible. In the case of solid-state EPR where the pulses applied cannot excite the entire width of the EPR spectrum, the spins are commonly divided into two types: those excited and observed by the microwave pulse are referred to as “A” spins, and the rest, termed “B” spins, are much more abundant. In general, at low temperatures, at which PD-EPR experiments are commonly carried out, there is no motion and the mechanisms contributing to decoherence for spin labels with S=1/2 are as follows (Salikhov et al., 1981; Tyryshkin et al., 2012; Mitrikas, 2023; Wilson et al., 2023; Eaton and Eaton, 2000). (i) Direct spin-lattice ( $T_{1}$ ) relaxation mechanism of the A spins, $T_{m,T_{1}}$ , provides the highest limit $T_{m}\leq 2T_{1}$ . (ii) Redistribution of resonance frequencies of dipole-coupled A spins due to microwave (mw) pulses leads to instantaneous diffusion (ID). (iii) Coupling between A spins and nearby B spins leads to spectral diffusion (SD). The latter can result from $T_{1}$ flips of the coupled B spins, referred to as indirect- $T_{1}$ , SD- $T_{1}$ , or energy-conserving pairwise B-spin flip-flops (SD-ee). All of these are concentration-dependent. (iv) Nuclear spin diffusion (NSD) arising from nuclear flip-flops caused by homonuclear couplings does not depend on the electron spin concentration but depends on the nucleus concentrations. (v) An admixture of tunnel states of methyl groups into the electron spin is mediated by the hyperfine coupling of methyl protons (Soetbeer et al., 2021a).

Gd(III) chelates are among the spin labels used for PD-EPR applications; they are beneficial for in-cell PD-EPR measurements because of their chemical stability and high sensitivity at high frequencies ( >34 GHz, Q-band and W-band) owing to a narrow central transition and a relatively long phase memory time (Goldfarb, 2014; Giannoulis et al., 2021). Several studies have been dedicated to the dephasing mechanism of Gd(III) at low temperatures. Raitsimring et al. (2014) explored the phase relaxation of Gd(III)-DOTA as a representative of Gd(III) spin labels in the temperature and concentration ranges typically used for W-band double electron-electron resonance (DEER) measurements, which is the most widely applied PD-EPR experiment (Pannier et al., 2000). They found that, in addition to the mechanisms of phase relaxation known for nitroxide-based spin labels listed above, Gd(III) spin labels are subjected to an additional phase relaxation mechanism that features an increase in the relaxation rate from the center to the periphery of the EPR spectrum. It was suggested that this mechanism is due to transient zero-field-splitting (tZFS) fluctuations. This tZFS-induced phase relaxation mechanism becomes dominant (or at least significant) when all other phase relaxation mechanisms mentioned above are significantly suppressed by matrix (solvent) deuteration and low spin concentrations.

A quantitative analysis of Gd(III) Hahn echo decay was recently reported at 240 GHz (Wilson et al., 2023). Two complexes, Gd-DOTA ( D=700 MHz) and Gd-PyMTA ( D=1200 MHz), were studied in D_2_O / glycerol-d_8_. $T_{1}$ and $T_{m}$ were measured as a function of temperature and concentration. As expected, $T_{1}$ was found to be concentration-independent, whereas $T_{m}$ was dependent. Interestingly, the Hahn echo decay from which $T_{m}$ was derived could be fitted by an exponential decay instead of the stretched exponent needed at Q-band (Soetbeer et al., 2021b). A careful analysis of the temperature and concentration dependence data and the associated $T_{1}$ values gave the relative contributions of the various decoherence mechanisms. The concentration-independent mechanism was found to be the direct- $T_{1}$ mechanism, and a concentration- and temperature-independent mechanism was assigned to weak coupling between electron spins and the presence of an ensemble of nuclear spins. As the solvent was fully deuterated, these could be protons on the Gd(III) complex. In this respect, it has been shown that, while deuteration of nitroxide spin labels did not increase $T_{m}$ , for trityl labels, it did (Soetbeer et al., 2021b).

Qualitative studies of several Gd(III) complexes, with axial parameters D , of the ZFS in the range 560–2000 MHz investigated the effect of solvent deuteration at Q-band (Garbuio et al., 2015) and reported that, in a protonated matrix, $T_{m}$ is dominated by NSD. It was also suggested that, in fully deuterated solvents, the phase relaxation is dominated by tZFS and perhaps ligand hyperfine-driven mechanisms, but no quantitative analysis of the data was presented. The decoherence behavior of Gd(III) complexes was further investigated under dynamical decoupling (DD) conditions. DD is a control strategy to protect quantum states from decoherence, which is achieved by applying a sequence of carefully designed control pulses that counteract unwanted interactions with the environment, effectively “decoupling” the system from environmental noise (Suter and Álvarez, 2016). CP (Carr–Purcell) and CPMG (Carr–Purcell–Meiboom–Gill) echo trains are such sequences. DD acts as a filter between the spin system and the environment, and the pulse spacing determines the characteristics of this filter. Short delays compared to the correlation times of the environmental fluctuations increase the coherence time of the system and, typically, relaxation caused by electron-electron spectral diffusion and NSD can be suppressed (Soetbeer et al., 2021b; Soetbeer et al., 2018). Recent measurements on a single crystal of Gd(III)-doped Y(trensal) carried out at X-band frequencies showed that CPMG with 120 refocusing pulses suppressed NSD efficiently and increased $T_{m}$ considerably, depending on the transition probed and the crystal orientation (Hansen et al., 2024).

Three Gd(III) complexes with D values of 485–1861 MHz were studied at Q-band in protonated and deuterated solvents, H_2_O / glycerol and D_2_O / glycerol-d_8_, and at a low concentration to suppress the ID and SD mechanisms (Soetbeer et al., 2021b). As expected, the solvent deuteration increased the decay time considerably, and the data could be well-fitted with a single stretched exponential decay function (SE model). Furthermore, for Gd-DOTA-M in deuterated solvents, DD (CP with up to five refocusing pulses) did not generate a significant increase in the decay time, and it was suggested that coherence losses of unknown origin, probably the ZFS-driven mechanism, which the DD cannot refocus, counteracted the decoupling efforts (Soetbeer et al., 2021b). Interestingly, measurements of a protein singly spin-labeled with Gd-DOTA-M in a deuterated solvent increased the echo decay rate approximately 3-fold as compared to the bare spin label in the same solvent. This 3-fold increase could be counteracted by DD with two to three pulses, achieving overall longer coherence survival than any DD trace of the fully protonated sample (Soetbeer et al., 2021b). These results showed that the protein's protons affect the phase relaxation of the Gd(III) spin label. Thus, one would expect that deuteration of the protein should help reduce the decoherence.

The $T_{1}$ values of Gd(III) complexes in solution are relatively short and therefore are expected to affect the Gd(III) phase relaxation. For example, the Gd(III) ruler with a PyMTA chelate with a distance of 3.4 nm have at W-band $T_{1}$ values in the range 80–11 µ s in the temperature range 6–30 K (Seal et al., 2022; Razzaghi et al., 2014). For the same type of ruler with distances of 2.1 and 6 nm, a $T_{1}$ value of ∼ 30 µ s was reported at 10 K (Mocanu et al., 2025). The reported $T_{1}$ values of the spin label BrPsPy-DO3A-Gd(III) in the temperature range 6–40 K are 132–9 µ s (Seal et al., 2022). At Q-band, the $T_{1}$ values are longer than at W-band; the complexes of the [GdIII(NO3Pic)] family, which have a small ZFS of D∼ 500 MHz and $T_{1}$ values in the range 190–200 µ s, were reported (Ossadnik et al., 2023).

**Figure 1:**
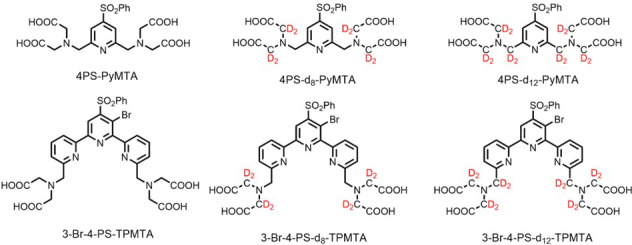
Deuterated chelates for Gd(III) that can be attached further to cysteine residues in a protein for labeling.

In the present work, we explore whether deuteration of the Gd(III) chelate and the protein can further extend $T_{m}$ , along with the effects of concentrations, temperature, and field position within the Gd(III) EPR spectrum. This is done for Hahn echo decay measurements, and the potential of DD to extend it further is also examined. We studied two Gd(III) spin-label complexes, PyMTA (Gd-PyMTA) and TPMTA (Gd-TPMTA) (Fig. 1), with different degrees of deuteration. These two complexes have very different D values (1200 vs. ∼ 4000 MHz). All measurements were carried out at W-band (95 GHz), and the complexes were dissolved in D_2_O / glycerol-d_8_, thus serving as a reference for the longest possible $T_{m}$ . These are then compared to the phase relaxation of these labels when conjugated to a protein. We found that the protons at a distance shorter than 5 Å from Gd(III) do not contribute to NSD and that the protein's deuteration did not significantly prolong $T_{m}$ . The primary mechanisms contributing to the phase relaxation in deuterated solvents and low concentrations are the direct- $T_{1}$ and tZFS mechanisms.

## 2 Experiments

### 2.1 Synthesis of spin labels

**Scheme 1:**
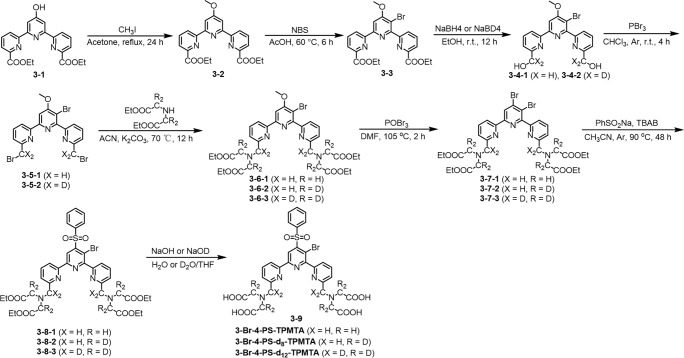
Synthesis of 3-Br-4-PS-(d_*n*_)-TPMTA spin labels.

The synthesis of spin labels, protein expression, and labeling is described in detail in the Supplement. In brief, the 4PS-(d_*n*_)-PyMTA labels were synthesized according to the reported procedure (Montgomery et al., 2017; Wang et al., 2019; Li and Byrd, 2022; Yang et al., 2015) and 3-Br-4-PS-(d_*n*_)-TPMTA labels were synthesized in the following eight steps (Scheme 1): 1 – methylation of (3-1) with CH_3_I and K_2_CO_3_ in acetone and purification via column chromatography (82 % yield); 2 – bromination of (3-2) with N-bromusuccinimide (NBS) in acetic acid at 60 °C and chromatography purification (92 % yield); 3 – reduction of (3-3) with NaBH_4_/NaBD_4_ in ethanol, yielding a yellow solid; 4 – reaction of (3-4) with PBr_3_ in CHCl_3_, followed by neutralization and extraction; 5 – substitution of (3-5) with diethyl iminodiacetate in acetonitrile at 70 °C, yielding a yellow solid; 6 – reaction of (3-6) with POBr_3_ in N,N-dimethylformamide (DMF) at 105 °C and purification; 7 – reaction of (3-7) with sodium benzene sulfinate and tetrabutylammonium bromide (TBAB) in acetonitrile at 90 °C; and 8 – hydrolysis of (3-8) with NaOH / NaOD in THF / H_2_O, followed by acidification, resulting in (3-9) as a yellow solid. The synthesis of 4PS-5-Br-6PCA-(d_*n*_)-DO3A-Gd(III), which is a new spin label, is described in detail in the Supplement. Mass spectra of all of the labels are shown in Figs. S1–S3.

### 2.2 Protein purification, spin labeling, and EPR sample preparation

The D39C / E64C construct of ubiquitin (ubi) was used in this study. The steps for deuterated protein expression were performed according to previous reports (Li and Byrd, 2022).

#### 2.2.1 Protein labeling with 4PS-5-Br-6PCA-(d_*n*_)-DO3A-Gd(III)

0.2 mM 100 µ L purified protein (^1^H^14^N ubi D39C / E64C or ^2^H^15^N ubi D39C / E64C) was incubated with 0.4 mM tris(2-carboxyethyl)phosphine (TCEP) in 20 mM Tris-HCl at pH 8.5 and then treated with 10 equivalents of labels at 30 °C for 12 h. The reaction progress was monitored by ESI-Q-TOF mass spectrometry. The excess label was removed using a PD-10 desalting column (GE Healthcare Biosciences). The ligation products were freeze-dried for subsequent experiments.

#### 2.2.2 Protein labeling with 4PS-PyMTA or 3-Br-4PS-TPMTA

The ligation of the target protein to 4PS-PyMTA was carried out according to the previous report (Yang et al., 2019). The 0.2 mM 100 µ L purified protein in 20 mM Tris-HCl at pH 8.5 was mixed with 0.4 mM TCEP and then treated with 10 equivalent labels at 30 °C for 12 h. After the reaction was completed, the sample was filtered through a PD-10 desalting column to remove the excess label. The protein PyMTA was mixed with 2.5 equivalents of Gd(NO_3_)_3_ in a 20 mM 2-morpholinoethanesulphonic acid (MES) buffer at pH 6.5. The excess metal ion was removed using a Millipore concentrator (3 kDa cutoff). Similarly, 3-Br-4PS-TPMTA was conjugated to the target protein using the same procedure, and the deuterated label were ligated to the target protein as with the non-deuterated label. The mass spectra of the labeled proteins are shown in Figs. S4–S7.

For pulsed EPR measurements, Gd-PyMTA and Gd-TPMTA solutions in the concentration range 0.03–0.2 mM were dissolved in 50:50 v/v D_2_O / glycerol-d_8_. The spin-label protein conjugates were lyophilized and redissolved in 15 mM HEPES-D_2_O buffer (pD 7.2) with 20 % glycerol-d_8_ ( v:v ). The final concentration of proteins was 50 µ M, estimated from the absorbance at 280 nm using a Nanodrop spectrophotometer (Thermo Science). For EPR measurements, solutions (ca. 3 µ L) were transferred to quartz capillaries (0.6 mm ID × 0.84 mm OD) and sealed at one end with crytoseal.

### 2.3 Spectroscopic measurements

Pulsed EPR and ENDOR measurements were performed using two home-built W-band pulsed EPR spectrometers equipped with cylindrical TE_011_ cavities and Helmholtz radiofrequency (RF) coils (Gromov et al., 1999). The first spectrometer has a solenoid superconducting magnet (Cryomagnetics, Inc.), a 3 W pulsed microwave power amplifier (QPP95013530, Quinstar), and a pulsed 2 kW RF amplifier (BT02000-GammaS, TOMCO). The second spectrometer has a 0–5 T cryogen-free magnet with an integrated variable temperature unit and a 300 mT sweep coil (J3678, Cryogenic Ltd.) (Feintuch et al., 2011) and is equipped with a 2 W pulsed microwave power amplifier (QPP95023330-ZW1, Quinstar). Temperature (below 15 K) and field dependencies of Gd-TPMTA were determined using the second spectrometer due to its wide temperature and magnetic field ranges.

Echo-detected EPR (ED-EPR) spectra were recorded employing the Hahn echo sequence ( π/2 – τ – π – τ –echo) and measuring the echo intensity as a function of the magnetic field. The π pulse duration was 28–30 ns, τ was 500 –600 ns, and a repetition time of 1 ms was employed. Echo decays as a function of τ were measured by setting the magnetic field to a position within the ED-EPR spectrum and the experimental parameters described above. The Carr–Purcell (CP) experiments were carried out using the π/2 –( τ/n – π – τ/n )_*n*_–echo sequence with a 2^*n*^-step phase cycle employed to filter out all additional echoes except the refocused ones (Soetbeer et al., 2018). For these experiments, n ranged from 1 to 5, with varying τ values for each value of n and measuring the intensity of the last echo as a function of τ . Additionally, a full CP train $\pi /2_{x}$ –( τ – $\pi_{x}$ – τ –echo– τ – $\pi_{-x}$ – τ –echo)_*n*_ (Mentink-Vigier et al., 2013) was applied with a two-step phase cycle on the first π/2 pulse, with a constant τ in the range 280–800 ns, and the intensity of each echo was measured, typically 2n∼ 140. $T_{1}$ measurements were performed using the inversion recovery sequence π – $t_{\mathrm{wait}}$ – π/2 – τ – π – τ –echo, with varying $t_{\mathrm{wait}}$ .

Mims ENDOR spectra were recorded on the first spectrometer at 10–20 K and a magnetic field corresponding to the maximum echo intensity using the sequence π/2 – τ – π/2 – T ( $\pi_{\mathrm{RF}}$ )– π/2 – τ –echo–[ $\tau_{2}$ – Π – $\tau_{2}$ –echo– $\tau_{2}$ –(– Π )– $\tau_{2}$ –echo]_*n*_ with a four-step phase cycle and five CP echoes with $\tau_{2}=600$ ns for detection, which was optimized for the best signal-to-noise ratio (Mentink-Vigier et al., 2013). The RF frequency was varied randomly (Epel et al., 2003). The experimental parameters for the Mims ENDOR spectra were T=42 µ s and τ , varying from 280 to 600 ns. RF power was adjusted to yield the desired $\pi_{\mathrm{RF}}$ pulse length (40 µ s) using a Rabi nutation sequence π/2 – τ – π/2 – T ( $t_{\mathrm{RF}}$ )– π/2 – τ –echo with a constant mixing time, T , of 100 µ s and a varying RF pulse length, $t_{\mathrm{RF}}$ .

### 2.4 Simulations of the ED-EPR spectra

ED-EPR spectra were simulated using the solid-state simulation function “pepper” of the EasySpin program package (version 6.0.0-dev.50) (Stoll and Schweiger, 2006). The distributions of ZFS parameters were considered using a built-in EasySpin functionality (DStrain parameter; the distributions in E and D were treated as uncorrelated), and the Boltzmann thermal polarization of the electron spin levels at W-band at the temperature of the experiment was taken into account. A Gaussian line shape with 0.1 mT width was used in the simulations. To account for the differences in turning angles for different electron spin manifolds in a pulsed ED-EPR experiment, intensities of individual transitions $|m_{S}\rangle ↔|m_{S}+1\rangle$ were re-normalized according to ${\mathrm{sin}}^{3}\left(\pi \alpha /2\right)\cdot \alpha^{-1}$ (Raitsimring et al., 2013), where $\alpha =\sqrt{S\left(S+1\right)-m_{S}\left(m_{S}+1\right)}/\sqrt{S\left(S+1\right)+0.25}$ and S=7/2 . This approach still does not consider the difference in phase memory times of different electron spin manifolds, which is minor for the short inter-pulse τ delays used in the ED-EPR sequence (500–600 ns). The optimal values of the parameters were determined by nonlinear least-squares fitting.

**Figure 2:**
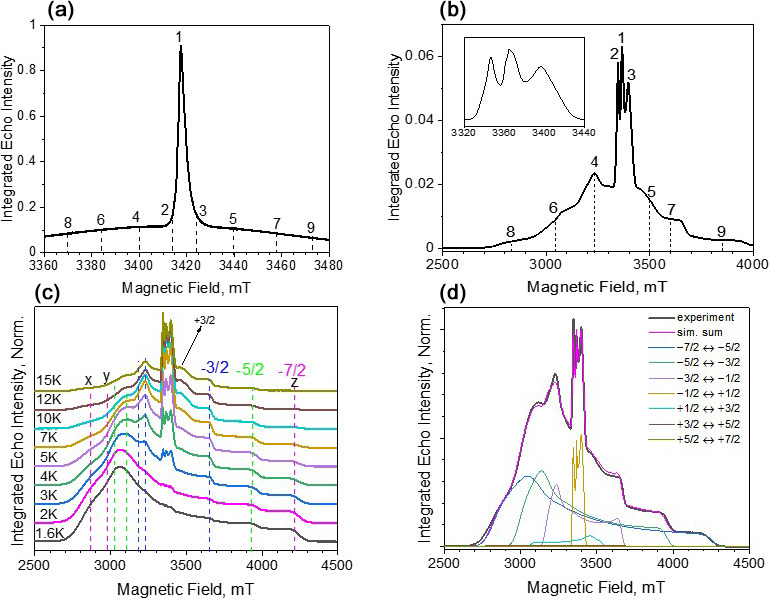
ED-EPR spectra (10 K) of Gd-PyMTA **(a)** and Gd-TPMTA **(b)**. The central transition of Gd-TPMTA is shown in the inset on the right with an extended scale. The different numbers indicate the positions at which relaxation measurements took place. **(c)** Temperature-dependent ED-EPR spectra of Gd-TPMTA. The positions of the x , y , and z singularities of the powder patterns of the various transitions are indicated by dashed lines according to the color code: blue for -3/2→-1/2 , green for -5/2→-3/2 , and magenta for -7/2→-5/2 . **(d)** Simulations of the spectrum in panel **(c)** recorded at 5 K: the simulation parameters are given in the text.

## 3 Results and discussion

### 3.1 Deuterated Gd(III) spin labels

#### 3.1.1 ED-EPR and ENDOR spectra

Before proceeding with the relaxation measurements, we carried out spectroscopic characterizations of the samples. The W-band ED-EPR spectra of Gd-PyMTA and Gd-TPMTA, recorded at 10 K, are shown in Fig. 2a, b. The spectrum of Gd-PyMTA is typical for Gd(III) spin labels with a moderate ZFS: ( D=1200 MHz)^27^ in frozen solutions where the $m_{S}=-1/2$ to $m_{S}=1/2$ CT dominates and appears as an intense peak superimposed on a broad featureless background, arising from all other transitions. The unresolved broad background results from a large distribution in the ZFS parameters D and E (Raitsimring et al., 2005). The spectrum of Gd-TPMTA is unusual; the central transition has a fine structure, and the broad background on which it is superimposed has clear singularities. This indicates that the ZFS is considerably larger than for Gd-PyMTA and that the distributions of D and E are smaller. To ease the assignment of the various features of the spectrum, we recorded the spectrum at lower temperatures, where the contribution of the CT decreases and those of the low-lying transitions |-7/2>→|-5/2> and |-5/2>→|-3/2> increase. The spectra are presented in Fig. 2c, with the annotations of the powder pattern's x , y , and z singularities corresponding to the various transitions. Simulations of the spectra presented in Fig. 2d gave D=4200 MHz, ΔD=390 MHz, E=440 MHz, and ΔE=370 MHz. We attribute the larger ZFS values and the smaller distributions to TPMTA offering nine optimal coordination sites for Gd(III) and holding it in a well-defined position. This is in contrast to PyMTA, which has seven coordination sites, and the other two are supplemented by water molecules.

**Figure 3:**
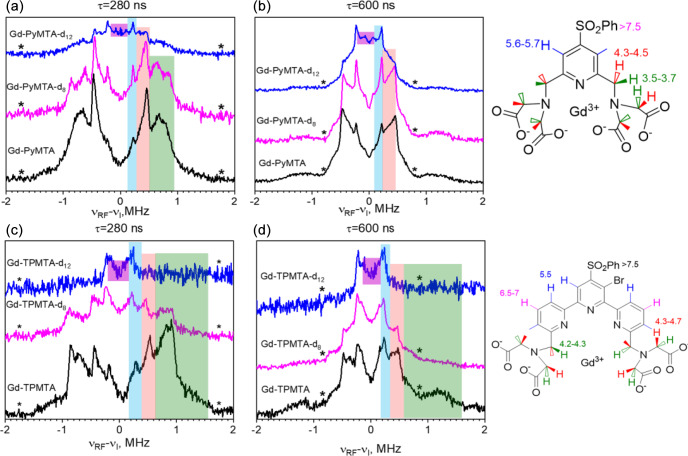
**(a, b)** Mims ENDOR spectra of Gd-PyMTA, Gd-PyMTA-d_8_, and Gd-PyMTA-d_12_ measured at the CT with two τ values (indicated in the figure). **(c, d)** Mims ENDOR spectra of Gd-TPMTA, Gd-TPMTA-d_8_, and Gd-TPMTA-d_12_ measured at the low field peak of the CT (3381 mT) with two τ values (indicated in the figure). The assignment of the signals is given by the colored stripes added to the spectra following the color code given in the complex structure shown on the right. The numbers next to the protons give the distances (Å) extracted from the ENDOR doublets' splitting under the assumption of purely dipolar couplings. The asterisks mark the positions of the blind spots.

Next, we carried out W-band Mims ENDOR measurements to test the efficiency of the deuteration and determine the hyperfine couplings of the different protons, which are important for identifying their potential contributions to decoherence by NSD. Figure 3a–b present the spectra of Gd-PyMTA, Gd-PyMTA-d_8_, and Gd-PyMTA-d_12_ measured with τ=280 ns to highlight the large ^1^H couplings and τ=600 ns to highlight the small ^1^H couplings.

**Figure 4:**
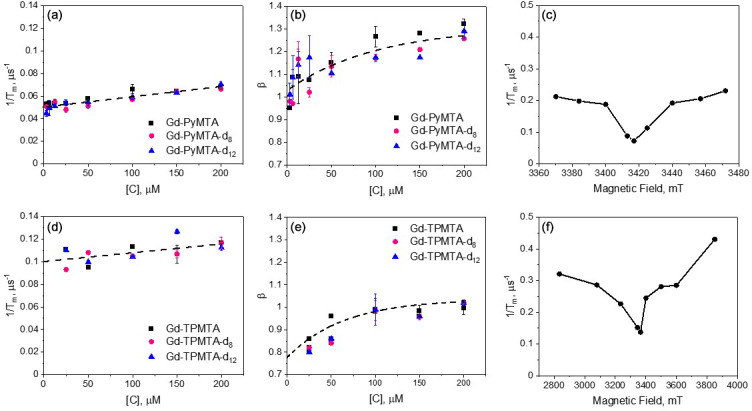
The dependence of $1/T_{m}$ and β measured at 10 K and the peak of the CT for the Gd-PyMTA variants **(a, b)** and Gd-TPMTA variants **(d, e)** measured at position 1 given in Fig. 2a and b, respectively. The dotted line in panels **(a)** and **(d)** is the linear fit with slopes of $9.07\times 10^{-5}\pm 8.2\times 10^{-6}$ and $7.79\times 10^{-5}\pm 2.9\times 10^{-5}$ ( µ s µ M)^−1^ and intercepts of 0.05 ± 0.01 and 0.100 ± 0.003 µ s^−1^ for Gd-PyMTA and Gd-TPMTA, respectively. The dotted lines in panels **(b)** and **(e)** were obtained with an exponential function $y=y_{0}+Ae^{-(\frac{x}{T})}$ to guide the eye. The parameters used were $y_{0}=1.3$ , A=-0.28 , and T=100.8 µ M and $y_{0}=1.04$ , A=-0.26 , and T=64.5 µ M for Gd-PyMTA and Gd-TPMTA, respectively. **(c, f)** The field dependence of $1/T_{m}$ for 200 µ M Gd-PyMTA **(c)** and 200 µ M Gd-TPMTA **(f)**, measured at 10 K.

For Gd-PyMTA-d_12_, the spectrum is dominated by the protons on the pyridine ring with a coupling $a_{⊥}=440$ kHz and the protons on the phenyl ring with $a_{⊥}=140$ –170 kHz, where $a_{⊥}$ is the principal perpendicular component of the hyperfine tensor. A comparison of the spectra of the three Gd-PyMTA samples shows some residual methylene protons. The ENDOR spectra of Gd-TPMTA, Gd-TPMTA-d_8_, and Gd-TPMTA-d_12_ are presented in Fig. 4c–d. In this case, the remaining protons of Gd-TPMTA-d_12_ are situated on three pyridine rings and are located 5.5 and 6.5–7 Å away from the Gd(III) with $a_{⊥}=170$ –440 kHz. Here, the deuteration efficiency was higher than that of Gd-PyMTA-d_12_, as we did not observe a significant contribution of residual methylene protons. A summary of the hyperfine couplings of the various protons in Gd-PyMTA and Gd-TPMTA is given in Table S1.

#### 3.1.2 Hahn echo decays

Hahn echo decays were measured for all samples and could be well-fitted with a single stretched exponential decay function (SE model) (Eq. 1):

1 $$y=A\times \mathrm{exp}\left[-{\left(\frac{2\tau }{T_{m}}\right)}^{\beta }\right].$$

For Gd-PyMTA, measurements were carried out in the range 3–200 µ M, and for Gd-TPMTA the range was 25–200 µ M; concentrations lower than 25 µ M were not tested because of sensitivity limits owing to the broader EPR spectrum of Gd-TPMTA. A few examples of echo decay data and their fits are shown in Fig. S8. The concentration dependence of $1/T_{m}$ measured at 10 K on the CT for the PyMTA variants is given in Fig. 4a. We chose 10 K because it is the optimal temperature for DEER measurements considering the populations of the CT and the $T_{m}$ temperature dependence (Goldfarb, 2014). We did not detect any apparent effect of the degree of deuteration on $T_{m}$ and β , both of which show a clear concentration dependence. This indicates that protons with hyperfine couplings in the range 1–2 MHz (distance <5 Å) do not lead to decoherence as they may be within the nuclear spin diffusion barrier (Wolfe, 1973).

The $1/T_{m}$ of Gd-PyMTA is linearly dependent on the concentration [ C ], and the intercept 0.05 µ s^−1^ gives $T_{m}$ (0) = 20 µ s: this is $T_{m}$ free of SD contributions. The dependence of β on [ C ] is not linear, reaching β=1 for [ C ] → 0 (Fig. 4b). The dependence of $1/T_{m}$ on the magnetic field within the EPR spectrum (10 K and [ C ] = 200 µ M), shown in Fig. 4c, reveals the same dependence as reported earlier (Raitsimring et al., 2014), where the central transition exhibits a longer $T_{m}$ , a characteristic of the tZFS mechanism (Raitsimring et al., 2014). The Hahn echo decay behavior of Gd-TPMTA, presented in Fig. 4d–f, was generally like that of Gd-PyMTA, disclosing no dependence on the deuteration levels. For Gd-TPMTA, the concentration dependence measurements were carried out at three field positions (1, 2, and 3; see Fig. 2a) within the CT, and the results of all three were practically the same: the data presented in Fig. 4d–f correspond to position 1. For Gd-TPMTA $T_{m}$ (0) = 10 µ s, the value of β is lower than for Gd-PyMTA and β=0.8 for [ C ] → 0. In general, β in the range 1–2.5 suggests the presence of a fast dephasing process attributed to SD or NSD (Salikhov et al., 1981; Eaton and Eaton, 2000), whereas β<1 is typical of slow processes or is a signature of the relaxation time distribution (Salikhov et al., 1981). Accordingly, we attribute the reduction in β with concentration to a reduction in the SD contribution, and the lower value of β at the low concentration limit of Gd-TPMTA is likely due to a larger, more extensive distribution of relaxation times. The latter arises from the larger ZFS and the significant contributions of transitions other than the CT at the CT field. We checked for the effect of ID for Gd-PyMTA with [ C ] = 200 µ M by measuring the echo decay as a function of the length of the second pulse and found a negligibly small contribution to $1/T_{m}$ and β (see Fig. S9). Therefore, we conclude that the contribution of ID to the concentration dependence of $1/T_{m}$ is minimal.

**Figure 5:**
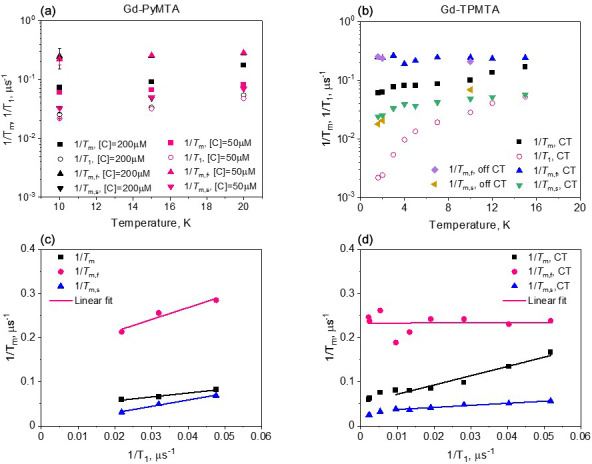
The temperature dependence of $1/T_{m}$ , determined with Hahn echo and full tbf(b). The dependence of $1/T_{m}$ , determined with Hahn echo and full CP train on $1/T_{1}$ for Gd-PyMTA **(c)** and Gd-TPMTA **(d)** along with the linear fit in the 6–20 K range.

Because the $T_{1}$ values of Gd(III) are relatively short and can influence its phase relaxation, we carried out $T_{1}$ and $T_{m}$ measurements at different temperatures for Gd-PyMTA and Gd-TPMTA. We only measured the non-deuterated variants as we did not see any effect of the complex deuteration on the echo decays. The $T_{1}$ values were determined from inversion recovery experiments and were analyzed using stretched exponents with values in the range 0.7–0.8 (see Fig. S10). Figure 5a shows the temperature dependences of $T_{1}$ and $T_{m}$ of 200 and 50 µ M Gd-PyMTA; as expected, $T_{1}$ is concentration-independent. For Gd-TPMTA a broader range of temperatures was accessed (1.6–15 K vs. 10–20 K), and the results are given in Fig. 5b. For both complexes, we found that, unlike $T_{m}$ , $T_{1}$ was independent of the field position within the EPR spectrum (Fig. S11); i.e., it is the same for all Gd(III) EPR transitions. For Gd(III), we must consider that it is not only the relaxation times that change with temperature but also the relative populations of the various transitions. Accordingly, changes in the levels' populations can influence the $T_{m}$ values measured at the CT. This effect is marginal for the temperature range explored for Gd-PyMTA but can be significant for Gd-TPMTA below 7 K.

To reveal the effect of $T_{1}$ on $T_{m}$ , we plotted $1/T_{m}$ vs. $1/T_{1}$ , and the results are shown in Figs. 5c–d. We observed a linear correlation for Gd-PyMTA (50 and 200 µ M). For Gd-TPMTA, where a wider range of temperatures was probed, a linear correlation was only observed for the 5–15 K range; below 5 K, $1/T_{m}$ is fairly constant, indicating that the contribution of $T_{1}$ to the phase relaxation is no longer significant.

The slopes of $1/T_{m}$ vs. concentrations [ C ] for both complexes are the same within the experimental error (Fig. 4). This indicates that the contribution of $1/T_{\mathrm{SD},\mathrm{ee}}$ is negligible; otherwise, a significant difference would be expected because of the much broader EPR spectrum of Gd-TPMTA. Therefore, we attribute the concentration dependence of $1/T_{m}$ to the indirect- $T_{1}$ , $T_{\mathrm{SD},T_{1}}$ mechanism, which is line-shape-independent. As the two, complexes have similar $T_{1}$ values, similar slopes are expected. We used the known expressions for $T_{\mathrm{SD},T_{1}}$ and $T_{\mathrm{ID}}$ to estimate their theoretical contributions (see Fig. S12 and the associated text) for Gd-PyMTA. We found the predicted contribution of $T_{\mathrm{ID}}$ to be negligible, consistent with our experimental results, and that the $T_{\mathrm{SD},T_{1}}$ calculated without any fitting parameters reproduces the experimental data reasonably (see Fig. S12), predicting a slope in the linear region of $1.2\times 10^{-4}$ µ s^−1^ µ M^−1^ compared to the experimental slope $0.9\times 10^{-4}$ µ s^−1^ µ M^−1^. The overestimated concentration dependence can result from the non-exponential behavior of the echo decay and the inversion recovery, i.e., β≠1 , and the underestimation of $T_{1}$ determined by the inversion recovery sequence. The contributions to $T_{m}$ (0) can be from the direct- $T_{1}$ relaxation, $T_{m,T_{1}}$ , residual NSD, and tZFS. As the contributions of spin diffusion, being either NSD or SD, to phase relaxation can be suppressed (refocused) by DD, we proceeded with measurements of $T_{m}$ using CP trains to further resolve the various contributions to phase relaxation.

#### 3.1.3 CP with n≤5

**Figure 6:**
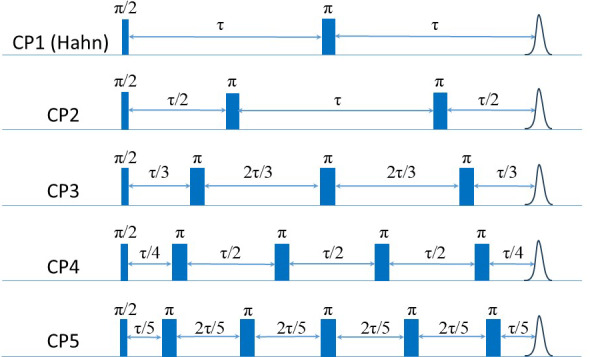
The CP n sequences for n=1 –5. n=1 corresponds to the Hahn echo.

**Figure 7:**
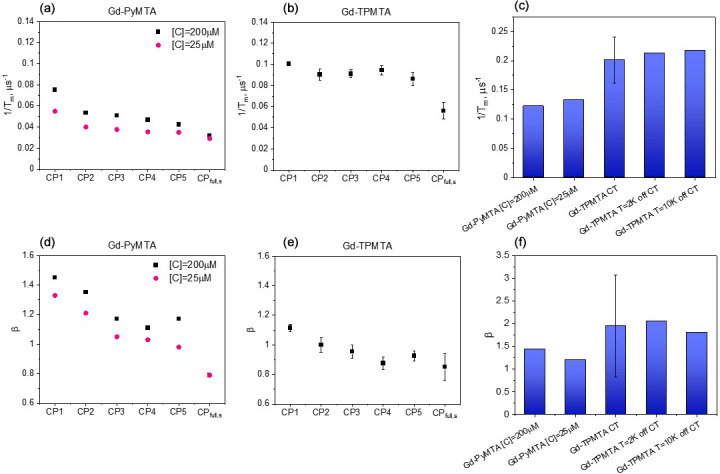
Comparison of $1/T_{m}$ measured at the CT and 10 K **(a, b)** and β **(d, e)**, determined by CP n with n=1 –5 and those of the slow component in the full CP train for Gd-PyMTA **(a, d)** and Gd-TPMTA **(b, e)**. **(c, f)** Comparison of the $1/T_{m}$ of the fast component in the full CP train **(c)** and β **(f)** for different complexes with different concentrations for Gd-PyMTA and Gd-TPMTA ([ C ] = 200 µ M) measured at different field positions within the EPR spectrum.

To resolve the potential contribution of NSD induced by the very weakly coupled protons on the labels to decoherence, we followed the approach used by Jeschke and coworkers (Soetbeer et al., 2018) and measured the intensity of the last echo as a function of the interval between the pulses for CP trains with two to five refocusing pulses (referred to as CP n ; see Fig. 6) while keeping the time between the first π/2 pulse and the last echo equal to 2τ for all n . Interferences from overlapping stimulated echoes can be eliminated by phase cycling up to n=5 ; beyond this, the phase cycle becomes too demanding (Soetbeer et al., 2018) (the phase cycles used are listed in Table S2). The resulting echo decays were analyzed using Eq. (1) (examples of fits are shown in Fig. S13), and the data from the protonated spin labels are given in Fig. 7a, where we plotted $1/T_{m}$ and β as functions of n , with n= 1 corresponding to the Hahn echo. We observed the same general behavior for 200 and 25 µ M Gd-PyMTA: an initial significant decrease in $1/T_{m}$ from n=1 to n=2 , followed by a mild change between n=2 and n=4 and leveling off at n=5 . β exhibits a monotonic decrease from n=1 to n=3 and levels off at n≥3 , where it reaches a value of 1–1.2. Nevertheless, the systematically larger $1/T_{m}$ for 200 µ M than that for 25 µ M and the reduction in the differences with n show that DD can suppress SD for the 200 µ M sample, though only partially for n=5 . The behavior of Gd-TPMTA was similar but less pronounced; $1/T_{m}$ decreases from n=1 to n=2 but then levels off, and β levels off between n=3 and n=4 and reaches 0.9. We find the suppression of NSD contributions to be less likely because Gd-TPMTA has more weakly coupled protons on the label, and therefore the effect should have been larger for Gd-TPMTA, but the opposite was observed. Such measurements, reported for Gd-DOTA-M in D_2_O : glycerol-d_8_ (25 µ M) at 10 K at Q-band, showed similar behavior, i.e., a mild decrease in $1/T_{m}$ and β (Soetbeer et al., 2021b). $T_{m}$ reached 40 µ s for n=5 for Gd-DOTA-M compared to 29 µ s for Gd-PyMTA at W-band.

#### 3.1.4 Full CP train

**Figure 8:**
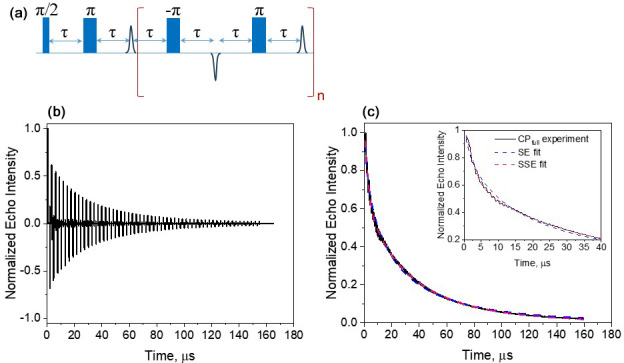
**(a)** The full CP train sequence ( n=137 and τ=290 ns) applied and **(b)** the resulting echo train for Gd-PyMTA, 50 µ M, 10 K, and the maximum of the CT. **(c)** The plot of the integrated echo intensity of the individual echoes as a function of time, with the data fit using a single stretched exponential and a sum of two stretched exponentials. The inset shows an expanded part of the trace, highlighting the fit differences. The fitting parameters were $T_{m}=32$ µ s, β=0.58 , and A=1.1 for the SE fit (Eq. 1) and $T_{m,f}=3.3$ µ s, $\beta_{f}=1.85$ , A=0.25 , $T_{m,s}=29.6$ µ s, and $\beta_{s}=0.78$ (Eq. 2) for the SSE fit.

The very mild effect of CP n with n=5 on the phase relaxation of both complexes prompted us to improve the effectiveness of the DD by applying a CP train pulse sequence with a constant inter-pulse delay τ (see Fig. 8a) and the shortest one available on our spectrometer (290 ns) to suppress potential contributions from fast processes to the phase relaxation. We refer to this as a full CP train. An example of the echo train produced by this sequence is given in Fig. 8b, and the plot of the echo intensities as a function of time is presented in Fig. 8c. In this case, the data could not be fitted satisfactorily with a single stretched exponent, and a sum of two stretched exponents – termed “SSEs” earlier (Soetbeer et al., 2018) – was used as follows:

2 $$y=A\times \mathrm{exp}{\left(-\frac{t}{T_{m,f}}\right)}^{\beta_{f}}+(1-A)\times \mathrm{exp}{\left(-\frac{t}{T_{m,s}}\right)}^{\beta_{s}},$$

where t is the time between the first π/2 pulse and the observed echo, and the subscripts “f” and “s” correspond to fast and slow processes.

As mentioned earlier, CP measurements with pulses that are not ideal (selective), because of their small bandwidth compared to the EPR spectral width, produce echoes that are not purely refocused but have contributions from stimulated echoes that decay with some combination of $T_{1}$ and spectral diffusion (Kurshev and Raitsimring, 1990; Mitrikas, 2023). To ensure that the observed SSE analysis is not a consequence of the contributions of such unwanted echoes, we performed a series of calculations presented in the Supplement (Figs. S14–S16). These show that the stimulated echo contribution leads to overestimation of $T_{m}$ by no more than 20 % and that a single stretched exponential function can fit the calculated echo intensities. To further ensure that the two observed components derived from the experimental results are not a consequence of artifacts in the applied pulse sequence, we carried out similar measurements on a nitroxide (MTSL) spin label in D_2_O : glycerol-d_8_ (25 µ M), and the results are shown in Fig. S17. In this case, the echo train could be fitted well with only one stretched exponent. Therefore, we concluded that the two resolved populations are intrinsic to the Gd(III) complexes studied and are not a consequence of the stimulated echo contributions or experimental artifacts.

**Figure 9:**
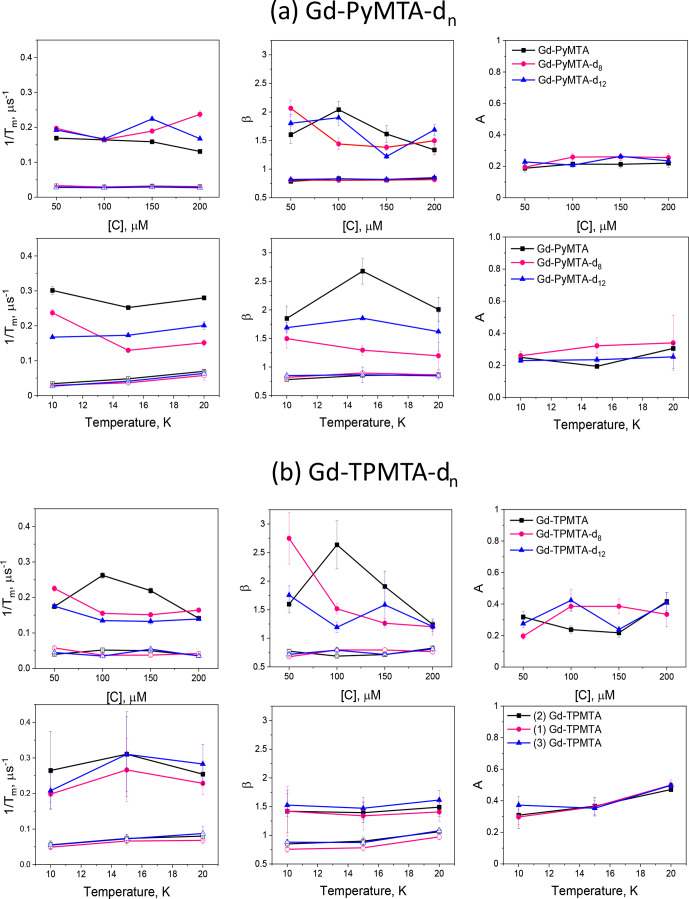
The dependence of the slow (open symbols) and fast (filled symbols) full CP train decay rates, the associated stretched exponent, and their relative population measured for **(a)** the Gd-PyMTA samples at the peak of the central transition as a function of concentration for 10 K and as a function of temperature for 200 µ M. **(b)** The Gd-TPMTA samples measured at position 1 in the central transition as a function of concentration (10 K) and as a function of temperature for 200 µ M at field positions 1, 2, and 3. The field positions are defined in Fig. 2a, b.

Figure 9 summarizes the dependence of the slow and fast CP decay rates, $1/T_{m,s}$ and $1/T_{m,f}$ , together with the associated $\beta_{s}$ and $\beta_{f}$ , on concentration and temperature for all Gd-PyMTA and Gd-TPMTA variants. The slow component of Gd-PyMTA, which has a contribution of 75 %–80 %, is concentration- and deuteration-independent, and at 10 K $T_{m}$ is 35 µ s, about twice as long as that measured by the Hahn echo decay (18 µ s) and well beyond the 20 % expected overestimation due to the selectivity of the applied pulses. The same holds for β , which is reduced to about 0.75. For comparison with the values obtained with CP1–CP5, we added the data to Fig. 7a, d. Interestingly, while CP5 could not eliminate the concentration dependence, the entire train did ( n∼140 ). Here, the $1/T_{m,s}$ values for 25 and 200 µ M Gd-PyMTA coincided. We should bear in mind that the full CP train and CP2–CP5 are different types of experiments; for the former, the number of π pulses and τ are held constant and the recorded signal is the intensity of the occurring refocused echo after each π pulse, and for the latter the number of pulses is constant, τ is varied, and the intensity of the last echo is measured. The short τ used in the full CP train seemed to refocus the SD contributions better than the CP5 experiment. We verified that, by increasing τ in the full CP train, the decay rate increased. There is an increase in $1/T_{m,s}$ with temperature, whereas $\beta_{s}$ and the relative population remain constant. For Gd-TPMTA, as for Gd-PyMTA, the slow component is not dependent on concentration or deuteration level. Still, there is an increase in $\beta_{s}$ and a decrease in its relative population with increasing temperatures. The values of $1/T_{m,s}$ and $\beta_{s}$ at 10 K are added to Fig. 7b, e for comparison with those obtained for CP1–CP5. The dependence of $1/T_{m,s}$ on the temperature and $1/T_{1}$ for the two complexes is shown in Fig. 5.

**Figure 10:**
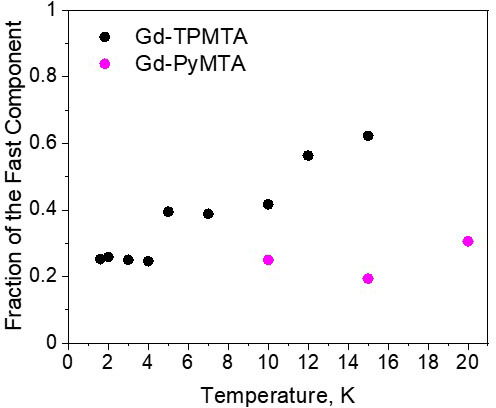
The dependence of the relative contribution of the fast component, measured at the CT (position 1), as a function of temperature for Gd-PyMTA (magenta, [ C ] = 200 µ M) and Gd-TPMTA (black, [ C ] = 200 µ M).

For the fast component, the spread of the data points is quite large for all variants, and no systematic variation with concentration or deuteration is observed. Here, $T_{m,f}$ is 3–6 µ s and $\beta_{f}=1.3$ –3; also, no temperature dependence was detected within the experimental error. A comparison of the various values of $1/T_{m,f}$ and $\beta_{f}$ for different samples and temperatures is given in Fig. 7c, f. The relative contribution of the two components is fairly constant in the temperature range tested for Gd-PyMTA, whereas for Gd-TPMTA the contribution of the fast component is constant for 1.6–4 K, and thereafter a significant increase with increasing temperature is observed in the range 6–15 K (Fig. 10). This trend seems to correlate with the relative intensity of the central transition (Fig. 2). Currently, we do not have an explanation for this behavior.

**Figure 11:**
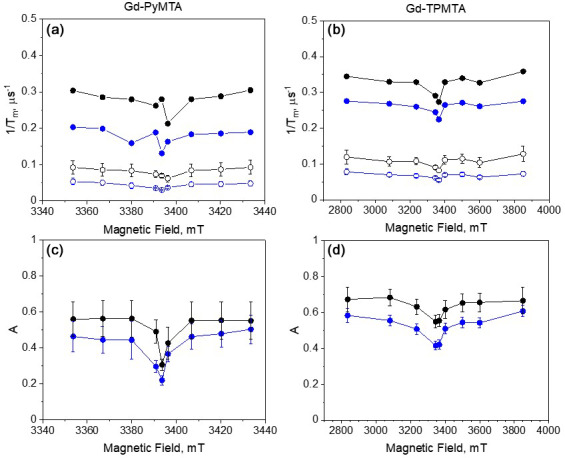
**(a)** The field dependence of $1/T_{m}$ for the slow (empty symbols) and fast (full symbols) components at 10 K (blue) and 20 K (black) of Gd-PyMTA ([ C ] = 200 µ M) and **(b)** Gd-TPMTA ([ C ] = 200 µ M). **(c)** The relative contribution of the fast component, A (see Eq. 2), for Gd-PyMTA and **(d)** Gd-TPMTA.

We also investigated the field dependence of the decay rates of two components at two temperatures, 10 and 20 K (Fig. 11). For the Hahn echo, we observed a clear enhancement of the decay rate outside the CT (see Fig. 4c, f); in contrast, the slow component showed a minimal change across the CT at both temperatures. Also, the difference between the CT and the other transitions was significantly weaker for the fast component than for the Hahn echo. Interestingly, the contrast between the CT and the other transitions is manifested in the two components' relative contributions. For both complexes, the contribution of the fast component is lower at the CT than outside the CT, and the contribution of the fast component increases with temperature in all of the fields.

From the full CP train measurements, we conclude that (i) two populations of spins with different dominating phase relaxation mechanisms are observed for the two complexes. (ii) Any residual SD_ee_ and SD_*T*_1__ contributions are suppressed under full CP train conditions. (iii) We tentatively assign the dominating mechanism that governs the slow-relaxing population to the direct- $T_{1}$ mechanism and tZFS for the fast-relaxing population. (iv) The relative contribution of the tZFS mechanism is lower at the central transition than at the other transitions. (v) Gd-TPMTA, which has a significantly larger ZFS than Gd-PyMTA, has a larger population dominated by the tZFS, which is also temperature-dependent. The temperature dependence seems to follow the relative intensity of the CT with temperature.

### 3.2 Influence of protein deuteration

**Figure 12:**
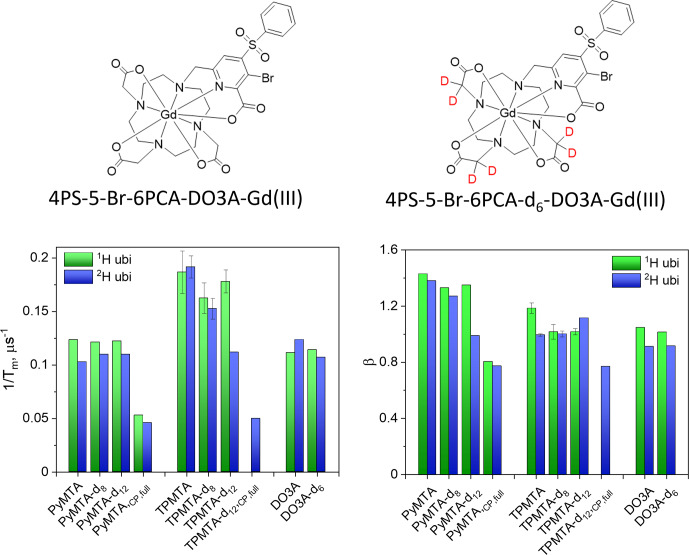
The structure of the Gd-DO3A label and the summary of the Hahn echo $1/T_{m}$ and β of ubiquitin labeled with Gd-PyMTA, Gd-TPMTA, and Gd-DO3A with various degrees of deuteration. $1/T_{m}$ and β of the slow component obtained from the full CP train measurements are presented as well.

For nitroxide spin labels, protein deuteration increases $T_{m}$ by a factor of ∼ 4 (Ward et al., 2010; Schmidt et al., 2016). To see whether Gd(III) spin labels experience the same effect, after exploring the phase relaxation behavior of the free Gd-PyMTA and Gd-TPMTA spin labels in deuterated solvents, we proceeded to examine their phase relaxation after their attachment to protonated and deuterated proteins in deuterated solvents. Ubiquitin D39C / E64C was labeled with Gd-PyMTA and Gd-TPMTA, producing doubly labeled proteins typically used for DEER applications. The concentrations were ∼ 25 and ∼ 50 µ M for Gd-PyMTA- and Gd-TPMTA-labeled ubiquitin, respectively. The Hahn echo decays were fitted using Eq. (1), as was done for the free labels (examples are shown in Fig. S18), and the results are summarized in Fig. 12 for measurements on the CT at 10 K. The attachment to ^1^H-ubiquitin increased $1/T_{m}$ by factors of 2.4 for Gd-PyMTA and 1.8–1.9 for Gd-TPMTA, with no significant effect on the degree of label deuteration. While protein deuteration led to a slight decrease in $1/T_{m}$ ( ∼ 10 %) for Gd-PyMTA, for Gd-TPMTA-labeled ubiquitin a significant effect was noticed only for Gd-TPMTA-d_12_, almost reaching the value of the free label. The dependence of β on protein deuteration is insignificant for Gd-TPMTA. In contrast, for Gd-PyMTA, a gradual decrease with the increased degree of the label deuteration is observed for the deuterated protein, where for Gd-PyMTA-d_12_ it reaches β=1 , as for the free label. A small effect of the protein deuteration was also observed for the new 4PS-5-Br-6PCA-DO3A-Gd(III), abbreviated as Gd-DO3A, labeled ubiquitin (50 µ M) (Fig. 12). The low impact of protein deuteration on the Gd(III) $T_{m}$ values compared to nitroxide can be attributed to Gd(III)'s much shorter $T_{1}$ , which provides the upper limit of $T_{m}$ ( $T_{m}\leq 2T_{1}$ ). A comparison of the $T_{m}$ and β values measured by Hahn echo decay for the free labels and the protein samples is given in Table S3.

To further explore the origin of the significant reduction in the phase relaxation rate while bound to protein and the small effect of protein deuteration, we carried out full CP train measurements on the protonated and deuterated protein samples for Gd-PyMTA and Gd-TPMTA. Like the free labels, the data could not be fitted well with one stretched exponent, and a sum of two such exponents was needed, one with a fast decay and the other with a slow decay (see Fig. S19). The results for the population with the slow decay for protonated and deuterated ubiquitin with protonated Gd-PyMTA are shown in Fig. 12. The difference between the protonated and deuterated proteins was small. As in the case of the free label, we observed a reduction by a factor of about 2 in $1/T_{m}$ for the slow population under CP train conditions as compared to the value for Hahn echo. Interestingly, $1/T_{m}$ in the free Gd-PyMTA in a deuterated solvent is smaller by a factor of about 2 compared to the protein value. The same behavior was observed for Gd-TPMTA.

What is the source of the faster phase relaxation in the protein compared to the free complex? It cannot be attributed to the direct- $T_{1}$ mechanism because of the similar $T_{1}$ values, i.e., 50.5 µ s for protein and 45.5 µ s for the free complex. One possibility could be NSD from the non-deuterated HEPES molecules used as a buffer, which results in 2.5 % protons in the solvent. Another possibility could be the lower amount of glycerol in the protein samples ( 8:2 v/v vs. 1:1 v/v for free Gd-PyMTA). To check this possibility, we prepared solutions of Gd-PyMTA in 15 mM HEPES in 8:2 v/v vs. 1:1 v/v D_2_O / glycerol-d_8_ and measured their Hahn echo decays at 10 K. The results, given in Fig. S20, show that the contribution of the protonated HEPES is small, but that of the lower amount of glycerol is significant because of enhanced instantaneous diffusion due to a poorer quality of the glass leading to higher local concentrations. These two effects only account for about 80 % of the shorter $T_{m}$ in the protein. An additional contribution can come from the fact that the proteins are doubly labeled; i.e., every Gd(III) center has a neighbor ∼ 4.2 nm away from it (see Fig. S21). Accordingly, its phase relaxation can be affected by indirect- $T_{1}$ due to the neighbor, which is concentration-independent. The relaxation of this neighbor is responsible for the Gd(III)–Gd(III) RIDME (Relaxation-Induced Dipolar Modulation Enhancement) PD-EPR experiment, where it undergoes single, double, and triple quantum flips during the mixing time (Razzaghi et al., 2014). In this case, mutual Gd(III) pair flip-flops can also induce relaxation (Tyryshkin et al., 2012).

## 4 Conclusions

In this work, we explored the mechanisms responsible for the phase relaxation of Gd(III) spin labels at 95 GHz, examining whether deuteration of the label or the protein can extend the phase relaxation. To resolve the relaxation mechanisms, we first studied the free label with different degrees of deuterations in a deuterated solvent and examined both concentration and temperature dependencies. We compared two labels with very different ZFSs, which helped resolve various relaxation mechanisms. $T_{m}$ was determined from both Hahn echo decay and CP echo trains. Our conclusions are as follows:

1. Protons with hyperfine couplings in the 1–2 MHz range, situated at distances <5 Å from the gadolinium ion, do not affect $T_{m}$ and are located within the nuclear spin diffusion barrier.
2. In the range 5–200 µ M, the concentration dependence of the free label is primarily determined by the indirect- $T_{1}$ -induced mechanism.
3. At the limit [ C ] → 0, the contributions to $T_{m}$ (0) can be the residual NSD of the protons on the pyridine and phenyl rings with hyperfine couplings below 0.4 MHz or residual protons in the deuterated matrix, tZFS, and direct $T_{1}$ . Since $T_{m}$ (0) for Gd-TPMTA is shorter by a factor of about 2 and $T_{1}$ values of both complexes are similar, we attribute the difference to the increased contribution of the residual NSD and tZFS. Gd-TPMTA has 12 weakly coupled protons vs. 7 for Gd-PyMTA, and its ZFS is significantly larger. In principle, it would be possible to predict the contribution of the above-mentioned weakly coupled protons and residual solvent protons to the Hahn echo decay using the analytical pair product approximation, which allows for computationally efficient simulations and provides a good prediction (Canarie et al., 2020; Jeschke, 2023). This, however, is beyond the scope of this paper.
4. The CP n measurements with n=2 –5 had a more substantial suppression effect on $T_{m}$ for Gd-PyMTA than on Gd-TPMTA, suggesting that it has contributions from SD due to electron-electron interactions and that NSD was not suppressed under these conditions ( n=5 ).
5. Full CP train measurements ( n∼140 ) resolved the presence of two populations: one with a slow phase relaxation and the other with a fast one. The dominating mechanism for the slow population is direct- $T_{1}$ . Its $T_{m}$ showed no concentration dependence and was longer by a factor of about 2 relative to the Hahn echo decay for both complexes while keeping their relative values. We tentatively assign the decrease in $1/T_{m,s}$ to full suppression of the residual indirect- $T_{1}$ -induced spectral diffusion and NSD mechanisms, made possible by the relatively short τ=290 ns used in the full train. This is supported by the more significant difference between n=5 and the full CP train for Gd-TPMTA, which has more distant protons.
6. For the fast relaxing population, $1/T_{m,f}$ is larger for Gd-TPMTA; therefore, we assign it to populations where the tZFS dominates, supported by its more extensive field dependence than $1/T_{m,s}$ .
7. Because of the relatively short $T_{1}$ and the contribution of the tZFS mechanism, protein deuteration does not significantly affect $T_{m}$ . The shorter $T_{m}$ for the doubly labeled proteins is attributed primarily to the lower glycerol amount in the sample and indirect- $T_{1}$ owing to the presence of a nearby Gd(III) neighbor.

The above shows that prolonging $T_{m}$ would mean increasing $T_{1}$ , which can be achieved by lowering the temperature. However, this will be at the expense of the CT population, thus reducing sensitivity in DEER measurements. Another option is to reduce the spectrometer frequency, which will cause broadening of the CT and impede sensitivity. Yet another way of increasing $T_{m}$ is to choose a label with a smaller ZFS (Ossadnik et al., 2025). A very small ZFS, however, introduces significant difficulties in analyzing DEER data for distances below 3 nm unless the excitation of the CT is avoided (Dalaloyan et al., 2015).

## Supplement

10.5194/mr-6-211-2025-supplement The supplement related to this article is available online at https://doi.org/10.5194/mr-6-211-2025-supplement.

## Data Availability

All data reported herein and the EasySpin scripts for simulation of the TPMTA label ED-EPR spectrum can be accessed at 10.5281/zenodo.15112855 (Edinach et al., 2025).

## References

[bib1.bib1] Canarie ER, Jahn SM, Stoll S (2020). Quantitative Structure-Based Prediction of Electron Spin Decoherence in Organic Radicals. J Phys Chem Lett.

[bib1.bib2] Dalaloyan A, Qi M, Ruthstein S, Vega S, Godt A, Feintuch A, Goldfarb D (2015). Gd(III)–Gd(III) EPR distance measurements–the range of accessible distances and the impact of zero field splitting. Phys Chem Chem Phys.

[bib1.bib3] Eaton SS, Eaton GR, Berliner LJ, Eaton GR, Eaton SS (2000). Distance Measurements in Biological Systems by EPR.

[bib1.bib4] Edinach E, Zhang X, Cui C-Y, Mitrikas G, Yang Y, Bogdanov A, Su X-C, Goldfarb D (2025). Zenodo [data set and code].

[bib1.bib5] Epel B, Arieli D, Baute D, Goldfarb D (2003). Improving W-band pulsed ENDOR sensitivity-random acquisition and pulsed special TRIPLE. J Magn Reson.

[bib1.bib6] Feintuch A, Shimon D, Hovav Y, Banerjee D, Kaminker I, Lipkin Y, Zibzener K, Epel B, Vega S, Goldfarb D (2011). A Dynamic Nuclear Polarization spectrometer at 95 GHz/144 MHz with EPR and NMR excitation and detection capabilities. J Magn Reson.

[bib1.bib7] Garbuio L, Zimmermann K, Häussinger D, Yulikov M (2015). Gd(III) complexes for electron–electron dipolar spectroscopy: Effects of deuteration, pH and zero field splitting. J Magn Reson.

[bib1.bib8] Giannoulis A, Ben-Ishay Y, Goldfarb D (2021). Characteristics of Gd(III) spin labels for the study of protein conformations. Meth Enzymol.

[bib1.bib9] Goldfarb D (2014). Gd^3+^ spin labeling for distance measurements by pulse EPR spectroscopy. Phys Chem Chem Phys.

[bib1.bib10] Gromov I, Krymov V, Manikandan P, Arieli D, Goldfarb D (1999). A W-band pulsed ENDOR spectrometer: Setup and application to transition metal centers. J Magn Reson.

[bib1.bib11] Hansen SH, Buch CD, Petersen JB, Rix M, Ubach I Cervera M, Strandfelt A, Winpenny REP, McInnes EJL, Piligkos S (2024). Probing decoherence in molecular 4f qubits. Chem Sci.

[bib1.bib12] Jeschke G (2023). Nuclear pair electron spin echo envelope modulation. J Magn Reson Open.

[bib1.bib13] Kurshev VV, Raitsimring AM (1990). Carr-Purcell train in the conditions of partial excitation of magnetic resonance spectrum. J Magn Reson.

[bib1.bib14] Li J, Byrd RA (2022). A simple protocol for the production of highly deuterated proteins for biophysical studies. J Biol Chem.

[bib1.bib15] Mentink-Vigier F, Collauto A, Feintuch A, Kaminker I, Le VT, Goldfarb D (2013). Increasing sensitivity of pulse EPR experiments using echo train detection schemes. J Magn Reson.

[bib1.bib16] Mitrikas G (2023). Long Electron Spin Coherence Times of Atomic Hydrogen Trapped in Silsesquioxane Cages. J Phys Chem Lett.

[bib1.bib17] Mocanu EM, Ben-Ishay Y, Topping L, Fisher SR, Hunter RI, Su X-C, Butler SJ, Smith GM, Goldfarb D, Lovett JE (2025). Robustness and Sensitivity of Gd(III)–Gd(III) Double Electron–Electron Resonance (DEER) Measurements: Comparative Study of High-Frequency EPR Spectrometer Designs and Spin Label Variants. Appl Magn Reson.

[bib1.bib18] Montgomery JRD, Lancefield CS, Miles-Barrett DM, Ackermann K, Bode BE, Westwood NJ, Lebl T (2017). Fractionation and DOSY NMR as Analytical Tools: From Model Polymers to a Technical Lignin. ACS Omega.

[bib1.bib19] Ossadnik D, Kuzin S, Qi M, Yulikov M, Godt A (2023). A Gd(III)-Based Spin Label at the Limits for Linewidth Reduction through Zero-Field Splitting Optimization. Inorg Chem.

[bib1.bib20] Ossadnik D, Qi M, Voss J, Keller K, Yulikov M, Godt A (2025). A Set of Three GdIII Spin Labels with Methanethiosulfonyl Groups for Bioconjugation Covering a Wide Range of EPR Line Widths. J Org Chem.

[bib1.bib21] Pannier M, Veit S, Godt A, Jeschke G, Spiess HW (2000). Dead-Time Free Measurement of Dipole–Dipole Interactions between Electron Spins. J Magn Reson.

[bib1.bib22] Raitsimring A, Astashkin AV, Enemark JH, Kaminker I, Goldfarb D, Walter ED, Song Y, Meade TJ (2013). Optimization of Pulsed-DEER Measurements for Gd-Based Labels: Choice of Operational Frequencies, Pulse Durations and Positions, and Temperature. Appl Magn Reson.

[bib1.bib23] Raitsimring A, Dalaloyan A, Collauto A, Feintuch A, Meade T, Goldfarb D (2014). Zero field splitting fluctuations induced phase relaxation of Gd^3+^ in frozen solutions at cryogenic temperatures. J Magn Reson.

[bib1.bib24] Raitsimring AM, Astashkin AV, Poluektov OG, Caravan P (2005). High-field pulsed EPR and ENDOR of Gd^3+^ complexes in glassy solutions. Appl Magn Reson.

[bib1.bib25] Razzaghi S, Qi M, Nalepa AI, Godt A, Jeschke G, Savitsky A, Yulikov M (2014). RIDME Spectroscopy with Gd(III) Centers. J Phys Chem Lett.

[bib1.bib26] Salikhov KM, Dzuba SA, Raitsimring AM (1981). The theory of electron spin-echo signal decay resulting from dipole-dipole interactions between paramagnetic centers in solids. J Magn Reson.

[bib1.bib27] Schmidt T, Wälti MA, Baber JL, Hustedt EJ, Clore GM (2016). Long Distance Measurements up to 160 Å in the GroEL Tetradecamer Using Q-Band DEER EPR Spectroscopy. Angew Chem Intl Ed.

[bib1.bib28] Seal M, Feintuch A, Goldfarb D (2022). The effect of spin-lattice relaxation on DEER background decay. J Magn Reson.

[bib1.bib29] Soetbeer J, Hülsmann M, Godt A, Polyhach Y, Jeschke G (2018). Dynamical decoupling of nitroxides in o-terphenyl: a study of temperature, deuteration and concentration effects. Phys Chem Chem Phys.

[bib1.bib30] Soetbeer J, Ibáñez LF, Berkson Z, Polyhach Y, Jeschke G (2021). Regularized dynamical decoupling noise spectroscopy – a decoherence descriptor for radicals in glassy matrices. Phys Chem Chem Phys.

[bib1.bib31] Soetbeer J, Millen M, Zouboulis K, Hülsmann M, Godt A, Polyhach Y, Jeschke G (2021). Dynamical decoupling in water–glycerol glasses: a comparison of nitroxides, trityl radicals and gadolinium complexes. Phys Chem Chem Phys.

[bib1.bib32] Stoll S, Schweiger A (2006). EasySpin, a comprehensive software package for spectral simulation and analysis in EPR. J Magn Reson.

[bib1.bib33] Suter D, Álvarez GA (2016). Colloquium: Protecting quantum information against environmental noise. Rev Mod Phys.

[bib1.bib34] Tyryshkin AM, Tojo S, Morton JJL, Riemann H, Abrosimov NV, Becker P, Pohl H-J, Schenkel T, Thewalt MLW, Itoh KM, Lyon SA (2012). Electron spin coherence exceeding seconds in high-purity silicon. Nat Mat.

[bib1.bib35] Wang G, Liang X, Chen L, Gao Q, Wang J-G, Zhang P, Peng Q, Xu S (2019). Iridium-Catalyzed Distal Hydroboration of Aliphatic Internal Alkenes. Angew Chem Intl Ed.

[bib1.bib36] Ward R, Bowman A, Sozudogru E, El-Mkami H, Owen-Hughes T, Norman DG (2010). EPR distance measurements in deuterated proteins. J Magn Reson.

[bib1.bib37] Wilson CB, Qi M, Han S, Sherwin MS (2023). Gadolinium Spin Decoherence Mechanisms at High Magnetic Fields. J Phys Chem Lett.

[bib1.bib38] Wolfe JP (1973). Direct Observation of a Nuclear Spin Diffusion Barrier. Phys Rev Lett.

[bib1.bib39] Yang Y, Wang J-T, Pei Y-Y, Su X-C (2015). Site-specific tagging proteins via a rigid, stable and short thiolether tether for paramagnetic spectroscopic analysis. Chem Comm.

[bib1.bib40] Yang Y, Yang F, Li X-Y, Su X-C, Goldfarb D (2019). In-Cell EPR Distance Measurements on Ubiquitin Labeled with a Rigid PyMTA-Gd(III) Tag. J Phys Chem B.

