# Supplementary material for: Can label or protein deuteration extend the phase relaxation time of Gd(III) spin labels?
Source: Magn Reson (Gott). 2025 Aug 12;6(2):211–28. doi: 10.5194/mr-6-211-2025 (PMC12753153; doi:10.5194/mr-6-211-2025)
Supplement: The supplement related to this article is available online at https://doi.org/10.5194/mr-6-211-2025-supplement. [file mr-6-211-2025-supplement.pdf]

Supplement of Magn. Reson., 6, 211–228, 2025
https://doi.org/10.5194/mr-6-211-2025-supplement
© Author(s) 2025. CC BY 4.0 License.

Open Access
MAGNETIC
RESONANCE

Supplement of

Can label or protein deuteration extend the phase relaxation time of Gd(III) spin labels?

Elena Edinach et al.

Correspondence to: Xun-Cheng Su (xunchengsu@nankai.edu.cn) and Daniella Goldfarb (daniella.goldfarb@weizmann.ac.il)

The copyright of individual parts of the supplement might differ from the article licence.

Contents

1. Experimental Section S2
1.1 Spin label synthesis S2
Synthesis of 4PS-5-Br-6PCA-(d)-DO3A-Gd^{3+}: S2
Synthesis of 4PS-(d)-PyMTA: S5
Synthesis of 3-Br-4-PS-(d)-TPMTA: S5
1.2 Expression and purification of non-deuterated and deuterated ubiquitin S7
1.3 Protein labeling S8
2. Mass spectrometry data S9
3. Mims ENDOR data S14
4. Examples of Hahn echo decay data S14
5. Testing for instantaneous diffusion S15
6. Inversion recovery data S16
7. Calculations of T_{ID} and T_{SD} S17
8. Examples of 2-5 CP echo decays. S18
9. Effect of stimulated echo on full CP train decay S20
10. Example of Hahn echo decays and CP data on labeled proteins. S24
11. Effect on HEPES and glycerol content of the Hahn echo decay of Gd-PyMTA S25
12. DEER data. S25
13. Table with overview of T_m and β values S26

1. Experimental Section

1.1 Spin label synthesis

Synthesis of 4PS-5-Br-6PCA-(d)-DO3A-Gd^{3+}:

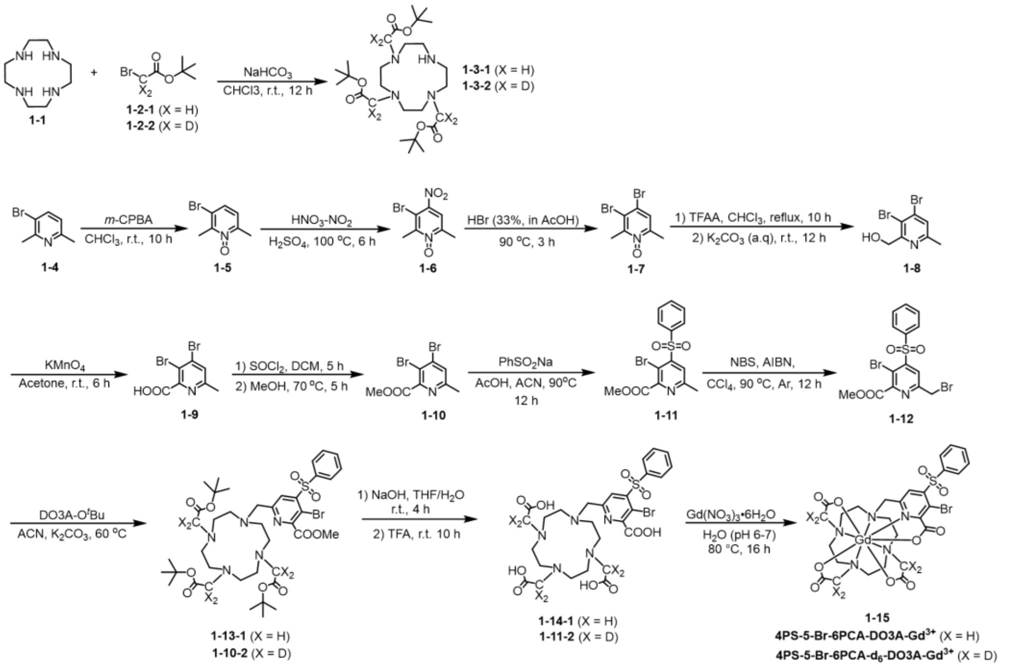

Synthesis of 1-5: The **1-2-2** and **1-3** were synthesized according to reported literature. (Montgomery et al., 2017; Wang et al., 2019; Li and Wong, 2004)

Synthesis of 1-5: To a solution of **1-4** (13.00ml, 100 mmol) in chloroform (50 mL), *m*-CPBA (110 mmol) was added slowly. The mixture was stirred at room temperature for 10 h. The formed precipitate was filtered and the filtrate was removed under vacuum. The crude product was purified by column chromatography to give the compound **1-5** as white solid (11.43 g, 57% yield). ^{1}H NMR (400 MHz, CDCl3) δ ppm: 7.39 (d, *J* = 8.2 Hz,1H), 7.05 (d *J* = 8.2 Hz, 1H), 2.75 (s, 3H), 2.52 (s, 3H).

Synthesis of 1-6: To compound **1-5** (9.56 g, 47.6 mmol) was added fuming nitric acid (30 mL) and concentrated sulfuric acid (25 mL) under 0 °C and mixture was stirred at 100℃ for 6 h. The mixture was cooled to room temperature and poured it into ice water. The water phase was extracted with DCM (3×100 mL). The organic phase was washed with saturate sodium dicarbonate solution and dried over sodium sulfate followed by removing the solvent under vacuum. The residue was purified by column chromatography to give the compound **1-6** as yellow solid (10.3 g, 88%). ^{1}H NMR (400 MHz, $CDCl_3$) δ ppm: 7.74 (1H, s), 2.81 (3H, s), 2.53 (3H, s); ^{13}C NMR (100 MHz, $CDCl_3$) δ ppm: 151.97, 148.78, 143.66, 118.50, 111.53, 18.73,

18.30.

Synthesis of 1-7: To hydrogen bromide in acetic acid (33%, 40 mL) compound **1-6** (9.34g, 40.27 mmol) was added. The mixture was stirred at 90 ℃ for 3 h and cooled to room temperature. The solution was poured into ice water and the pH was adjusted to 7 with sodium hydroxide. The water phase was extracted with DCM (3×100 mL). The organic phase was dried over sodium sulfate and filtered through celite. The residue was purified by column chromatography to give the compound **1-7** as yellow solid (10.6 g, 94% yield). ^{1}H NMR (400 MHz, $CDCl_3$) δ ppm: 7.47 (s, 1H), 2.84 (s, 3H), 2.45 (s, 3H).

Synthesis of 1-8: To a solution of **1-7** (6.0 g, 24.4mmol) in chloroform was added TFAA (10 mL) dropwise. Then the mixture was stirred at 65℃ for 10 h. The solvent was removed under vacuum followed by adding saturate sodium decarbonate and stirred at room temperature overnight. The water phase was extracted with DCM (3×100 mL). The organic phase was dried over sodium sulfate and filtered through celite. The residue was purified by column chromatography to give the compound **1-8** as a white solid (2.1 g, 35% yield). ^{1}H NMR (400 MHz, $CDCl_3$) δ ppm: 7.42 (1H, s), 4.84 (2H, d, J = 4.6 Hz), 4.30 (1H, t, J = 4.6 Hz), 2.69 (3H, s); ^{13}C NMR (100 MHz, $CDCl_3$) δ ppm: 159.74, 158.53, 116.43, 105.63, 63.73, 23.87.

Synthesis of 1-9: To a solution of **1-8** (8.75g, 31.25mmol) in acetone $KMnO_4$ was added. Then the mixture was stirred at room temperature for 6 h. Then the insoluble $KMnO_4$ was filtered through celite and the filtrate was concentrated under vacuum to afford the **1-9** as yellow solid (8.03 g, 88%). ^{1}H NMR (400 MHz, $CDCl_3$) *δ* ppm: 7.40 (s, 1H), 2.34 (s, 1H).

Synthesis of 1-10: To a solution of **1-9** (24.87g, 84.90mmol) in dichloromethane (200 mL), dichlorosulfoxide (61.59ml, 849.00mmol) was added. Then the reaction mixture was stirred at room temperature for 5 h. the solvent was removed under reduced pressure. Methanol (140 mL) was added at room temperature, and then the reaction mixture was stirred at 70 °C for 5 h. The mixture was concentrated under reduced pressure. The residue was purified by flash chromatography on silica gel to give **1-10** as a yellow solid (8.84 g, 40% yield). ^{1}H NMR (400 MHz, $CDCl_3$) *δ* ppm: 7.39 (s, 1H), 4.02 (s, 3H), 2.56 (s, 3H).

Synthesis of 1-11: The **1-10** was synthesized from **1-28** according to general method A. Light yellow oil (14.85g, 55% yield). ^{1}H NMR (400 MHz, $CDCl_3$) *δ* ppm: 8.19 (s, 1H), 7.98 (d, J = 8.7

Hz, 2H), 7.69 (t, J = 7.6 Hz, 1H), 7.58 (t, J = 7.1 Hz, 2H), 3.98 (s, 3H), 2.70 (s, 3H).

Synthesis of 1-12: The **1-11** (14.85 g, 40.25 mmol) was dissolved in 200 mL CCl_4, after the mixture was heated to 90 °C, the NBS (7.1g, 40.2 mmol) and AIBN (328 mg, 2.0mmol) was added portion-wise. After the TLC showed the full conversion, the reaction mixture was cooled to room temperature and filtered. The filtrate was concentrated and the crude product was purified by column chromatography to give the compound **1-12** as yellow solid (9.78 g, 54% yield). ^{1}H NMR (400 MHz, $CDCl_3$) δ ppm: 8.47 (s, 1H), 7.99 (d, J = 8.2 Hz, 2H), 7.72 (t, J = 7.2 Hz, 1H), 7.60 (t, J = 7.2 Hz, 2H), 4.62 (s, 2H), 4.00 (s, 3H).

Synthesis of 1-13: To a solution of **1-12** (0.25g, 0.56mmol) in acetonitrile (20 mL), DO3A-^{t}Bu (308 mg, 0.6mmol) and potassium carbonate (0.3g, 2.5 mmol) were added. The mixture was stirred at room temperature for 20 h. Then the mixture was filtered, and the solvent of the filtrate was removed under vacuum, the residue was purified on silica gel to give the **1-13** as yellow oil (277 mg, 56% yield). HRMS (ESI) calcd for $[C_{40}H_{60}BrN_5O_{10}S + H]^+$: 882.3317, found: 882.3328.

Synthesis of 1-14: To a solution of **1-13** (264 mg, 0.30mmol) in THF/H_2O (2:1, 15 mL) sodium hydroxide (16 mg, 0.4 mmol) was added slowly. The mixture was stirred at room temperature for 11 h. The concentrate HCl was added to adjust the pH to 3~4, and the solvent was removed. DCM/TFA (10 mL, 1:1) was added and the mixture was stirred for 0.5 h. The solvent was removed under vacuum to give the **1-14** as a white solid (202 mg, 93% yield). HRMS (ESI) calcd for $[C_{27}H_{34}BrN_5O_{10}S + H]^+$: 700.1283, found: 700.1286.

Synthesis of 4PS-5Br-6PCA-(d_6)-DO3A-Gd^{3+}: To a 50 mL hydrothermal reactor **1-14** (140 mg, 0.2 mmol) and deionized water (10 mL) were added. The pH was adjusted to 6-7, then the mixture was left to 80 °C for 20 h. After cooling down to room temperature, the residue was adjusted to pH 9.0 and the precipitate was filtered. The filtrate was adjusted to pH 5. The solvent was removed under vacuum, then methanol was added to resolve the target product. The precipitate was filtered and filtrate was removed to deliver the target product.

4PS-5Br-6PCA-DO3A-Gd^{3+}: (90% yield). HRMS (ESI) calcd for $[C_{27}H_{30}BrGdN_5O_{10}S + H]^+$: 855.0269, found: 855.0271.

4PS-5Br-6PCA-d_6-DO3A-Gd^{3+}: (86% yield). HRMS (ESI) calcd for $[C_{27}H_{24}D_6BrGdN_5O_{10}S + H]^+$:

861.0588, found: 861.0669

Synthesis of 4PS-(d)-PyMTA:

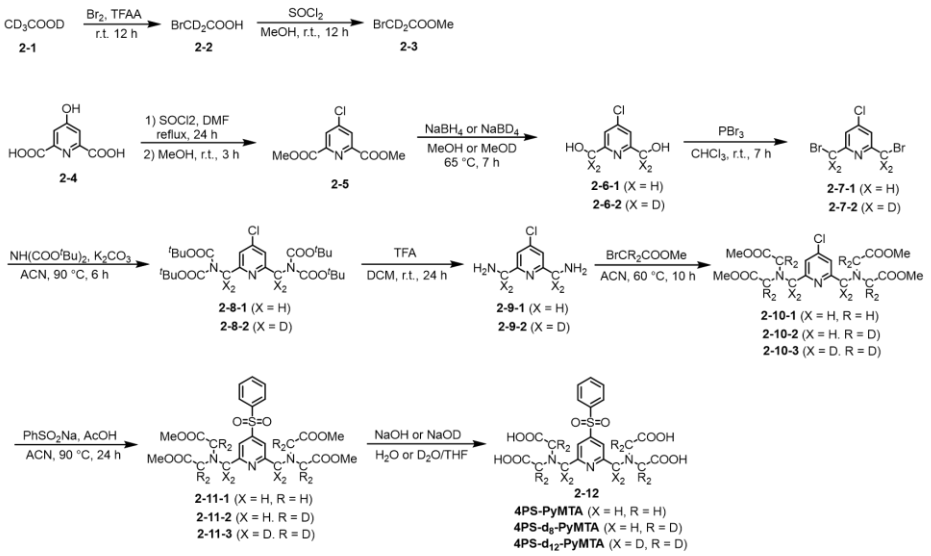

The 4PS-(d)-PyMTA were synthesized according to reported literature.(Montgomery et al., 2017; Wang et al., 2019; Yang et al., 2015)

4PS-d$_8$-PyMTA: HRMS (ESI) calcd for $[C_{21}H_{15}D_8N_3O_{10}S + H]^+$: 518.1679, found: 518.1685.

4PS-d$_{12}$-PyMTA: HRMS (ESI) calcd for $[C_{21}H_{11}D_{12}N_3O_{10}S + H]^+$: 522.1930, found: 522.1918.

Synthesis of 3-Br-4-PS-(d)-TPMTA:

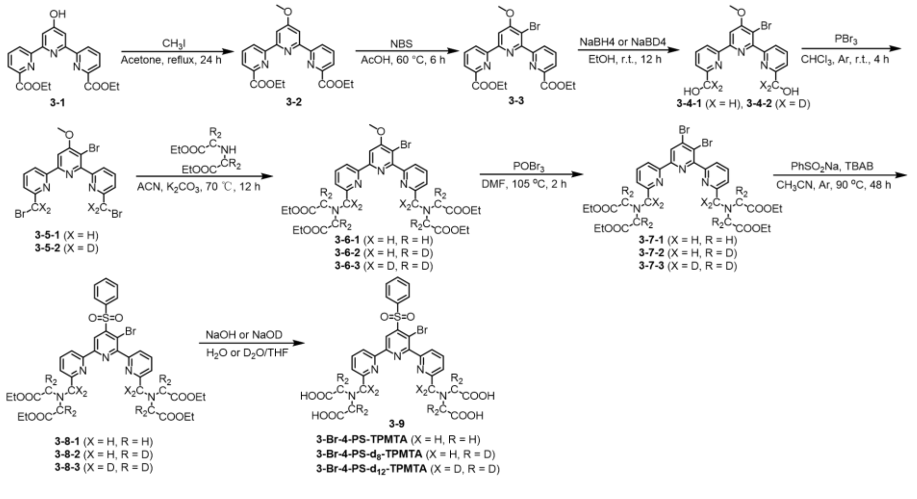

Synthesis of compound 3-2: The **3-1** was synthesized according to the reported literature (Kadjane et al., 2009). To a mixture of compound **3-1** (3.9 g, 10 mmol) and K_2CO_3 (2.7 g, 20 mmol) in acetone (50mL) CH_3I was added (2.1 g, 15 mmol), and the mixture was stirred at

room temperature for 24 h. Then the solvent was evaporated under reduced pressure. The obtained crude product was purified by column chromatography (DCM: MeOH 50:1) to deliver the compound **3-2** as a white solid (3.3 g, 82% yield). ^{1}H NMR (400 MHz, $CDCl_3$) δ 8.76 (dd, *J* = 7.9, 1.1 Hz, 1H), 8.16 (s, 1H), 8.13 (dd, *J* = 7.7, 1.1 Hz, 1H), 7.97 (t, *J* = 7.8 Hz, 1H), 4.51 (q, *J* = 7.1 Hz, 2H), 1.48 (t, *J* = 7.1 Hz, 3H). HRMS (ESI) calcd for $[C_{22}H_{21}N_3O_5 + H]^+$: 408.1554, found: 408.1553.

Synthesis of compound 3-3: To a solution of compound **3-2** (3.3 g, 8.2 mmol) in acetic acid (30 mL) NBS (4.3 g, 24.6 mmol) was added and the mixture was stirred under 60 °C for 4 h in the dark. After cooling to room temperature, the mixture was poured into ice water and neutralized with saturated K_2CO_3 solution. The residue was extracted with DCM (3x100 mL). The organic phase was dried over dried over Na_2SO_4 followed by filtering and removing under vacuum. Purification of the crude product was achieved by column chromatography (DCM : MeOH 100:1) to provide **3-3** as a white solid (3.6 g, 92% yield). ^{1}H NMR (400 MHz, $CDCl_3$) δ 8.66 (dd, *J* = 8.0, 1.1 Hz, 1H), 8.24 (dd, *J* = 7.8, 1.2 Hz, 1H), 8.15 (s, 1H), 8.14 (d, *J* = 1.1 Hz, 1H), 8.01 (t, *J* = 7.8 Hz, 1H), 7.97 – 7.87 (m, 2H), 4.18 (s, 3H), 2.97 (s, 6H). HRMS (ESI) calcd for $[C_{22}H_{20}N_3O_5Br + H]^+$: 486.0660, found: 486.0677.

Synthesis of 3-4: Compound **3-3** was dissolved in 250 ml EtOH, then $NaBH_4$ or $NaBD_4$ (5 eq) was added under ice water bath condition. The mixture was stirred at room temperature for 12 h. After quenching reaction with saturated $NaHCO_3$ solution, brown yellow filtrate was obtained by suction filtration. The filtrate was extracted with dichloromethane and dried over anhydrous sodium sulfate, filtered and dried to afford **3-4** as yellow solid.

Synthesis of 3-5: To a solution of **3-4** (1.02 g, 4.08 mmol) in 20 mL of $CHCl_3$, PBr_3 (1.20 mL, 12.25 mmol) was added at ice bath. The mixture was stirred under room temperature and argon atmosphere for 5 h. Then the solution was poured into the ice water after cooling to room temperature, The $NaHCO_3$ was added to adjust the pH to weak alkalinity and the mixture was extracted with DCM. The organic layer was dried over Na_2SO_4. The solvent was removed in vacuo and the obtained residue was purified by silica gel column chromatography to give the **3-5** as white solid.

To a solution of **3-5** in acetonitrile, diethyl iminodiacetate (3 eq) and potassium carbonate (4

eq) was added. The mixture was stirred at 70 °C for 12 h. Then the mixture was filtered, and the solvent of the filtrate was removed under vacuum, the residue was purified on silica gel to give the **3-6** as yellow solid.

Synthesis of 3-7: To a solution of compound **3-6** in dry DMF (30 mL) $POBr_3$ (5 eq) was added and the mixture was stirred under 105 °C for 2 h. After cooling to room temperature, the mixture was poured into ice water and neutralized with saturated K_2CO_3 solution. Then the residue was extracted with ethyl acetate. The organic phase was washed with brine and dried over Na_2SO_4 followed by filtering and removing under vacuum. Purification of the crude product was achieved by column chromatography to provide **3-7** as a white solid.

Synthesis of 3-8: Sodium benzene sulfinate (3 eq) and TBAB (0.5 eq) was added to a solution of compound **3-7** in dry acetonitrile. The mixture was stirred at 90 °C under argon for 12 h. After cooling to room temperature, the precipitate was filtered over celite. Then the solvent was evaporated under reduced pressure. The obtained crude product was purified by column chromatography to deliver the compound **3-8.**

Synthesis of 3-9: To a solution of compound **3-8** in THF/H_2O (D_2O) was added NaOH or NaOD (6 eq) and the mixture was stirred under room temperature for 4 h. THF was evaporate under vacuum and the residue was acidified 2 M HCl solution to pH 4-5 resulting in precipitate. Collecting and drying the precipitate to give the tag **3-9** as yellow solid

3-Br-4-PS-TPMTA: HRMS (ESI) calcd for $[C_{31}H_{28}BrN_5O_{10}S + H]^+$: 742.0814, 744.0793, found: 742.0811, 744.0798.

3-Br-4-PS-d$_8$-TPMTA: HRMS (ESI) calcd for $[C_{31}H_{20}D_8BrN_5O_{10}S + H]^+$: 750.1316, found: 750.1319.

3-Br-4-PS-d$_{12}$-TPMTA: HRMS (ESI) calcd for $[C_{31}H_{16}D_{12}BrN_5O_{10}S + H]^+$: 754.1567, found: 754.1562.

1.2 Expression and purification of non-deuterated and deuterated ubiquitin

The double mutations D39C/E64C of ubiquitin were used in this study. The cDNA of ubi was cloned into the pET28a vector containing an ampicillin resistance and T7 promoter. The plasmid was transformed in *E. coli* BL21 (DE3) CondonPlus strain for protein expression.

Expression of non-deuterated ubiquitin. Briefly, the cells were grown in 600 mL Luria broth

(LB) medium at 37°C until OD_{600} = 0.8, and then protein expression was induced by the addition of 1.0 mM isopropyl-beta-D-thiogalactopyranoside (IPTG) for overnight.

Expression of deuterated ubiquitin. The steps for deuterated protein expression were performed according to previous reports. (Li and Byrd, 2022). In M9 minimal medium, $^{15}NH_4Cl$ (98% ^{15}N) and ^{2}H-glucose (98% ^{2}H) were added as the sole source of nitrogen and carbon with D_2O (99.9% ^{2}H) as the solvent.

The bacterial cells were collected by centrifuge and lysed by sonication. Proteins were then purified by two column chromatography steps, DEAE column (DEAE Sepharose FF, Cytiva) and a size exclusion chromatography (HiLoad 16/600 Superdex 75, Cytiva). Pure ubiquitin fractions were determined by SDS-PAGE and ESI-Q-TOF. The molecule weights of ^{1}H ^{14}N ubiquitin D39C/E64C and ^{2}H ^{15}N ubiquitin D39C/E64C measured by mass spectrometry were 8527.3Da and 9107.6Da (all exchangeable protons are ^{1}H), respectively. Another molecular weight of 9218.7 observed in ^{2}H ^{15}N ubi D39C/E64C mass spectrum corresponds to the mass of ^{2}H, ^{15}N- ubiquitin D39C/E64C in which a few exchangeable ^{2}H were placed by ^{1}H. Assuming that the labeling efficiency of ^{15}N was 90%, the calculated deuterium labeling efficiency in ^{2}H ^{15}N ubiquitin D39C/E64C is approximately 94.4%.

1.3 Protein labeling

Protein labeling with 4PS-5-Br-6PCA-DO3A-Gd(III) or 4PS-5-Br-6PCA-d_6-DO3A-Gd(III). 0.2 mM 100 μL purified protein (^{1}H, ^{14}N ubiquitin D39C/E64C or ^{2}H, ^{15}N u ubiquitin D39C/E64C) was incubated with 0.4 mM tris(2-carboxyethyl)phosphine (TCEP) in 20 mM Tris-HCl at pH 8.5, and then treated with 10 equivalents tags at 30 °C for 12 h. The reaction progress was monitored by ESI-Q-TOF mass spectrometry. The excess tag was removed using a PD-10 desalting column (GE Healthcare Biosciences). The ligation products were freeze-dried for subsequent experiments.

Protein labeling with 4PS-PyMTA or 3-Br-4PS-TPMTA. The ligation of the target protein to 4PS-PyMTA were carried out according to the previous reports (Yang et al., 2019). 0.2 mM 100 μL purified protein in 20 mM Tris-HCl at pH 8.5 was mixed with 0.4 mM TCEP, and then treated with 10 equivalents of tags at 30 °C for 12 h. After the reaction was completed, the sample was filtered through PD-10 desalting column to remove the excess probe. The

protein-PyMTA was mixed with 2.5 equivalents $Gd(NO_3)_3$ in 20 mM MES buffer at pH 6.5. The excess of metal ion was removed using a Millipore concentrator (3 kDa cutoff). Similarly, 3-Br-4PS-TPMTA was conjugated to the target protein using the same procedure, and the deuterated tags were ligated to the target protein also in the same steps as the non-deuterated tag.

For pulse EPR measurements, the spin-labelled protein conjugates were lyophilized and redissolved in HEPES-D_2O buffer (pD 7.2) with 20% glycerol-d_8 (v:v). The final concentration of proteins was 50 mM determined by UV280 with a Nanodrop one[c] (Thermo Science).

2. Mass spectrometry data

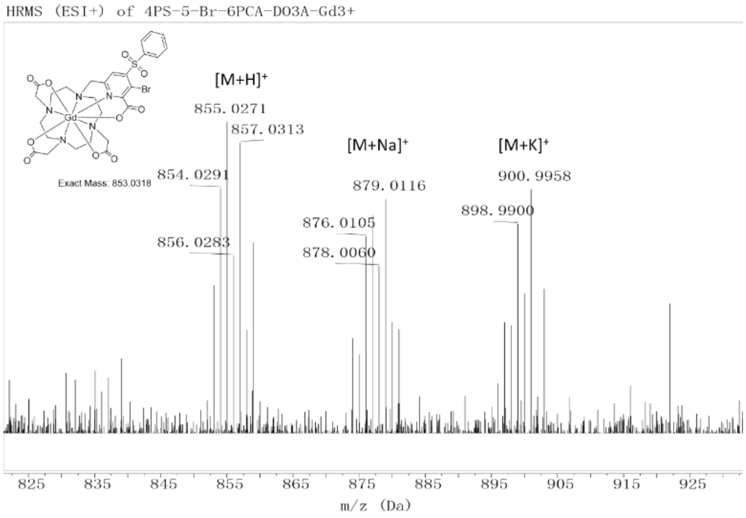

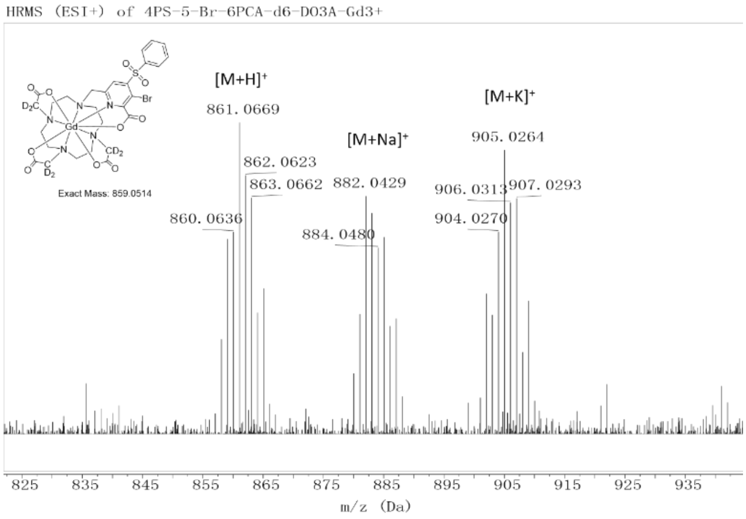

Figure S1. *High-resolution mass spectra of 4PS-5-Br-6PCA-DO3A-Gd(III) and 4PS-5-Br-6PCA-d_6-DO3A-Gd(III). The spectra were recorded on an ESI-Q-TOF mass spectrometer.*

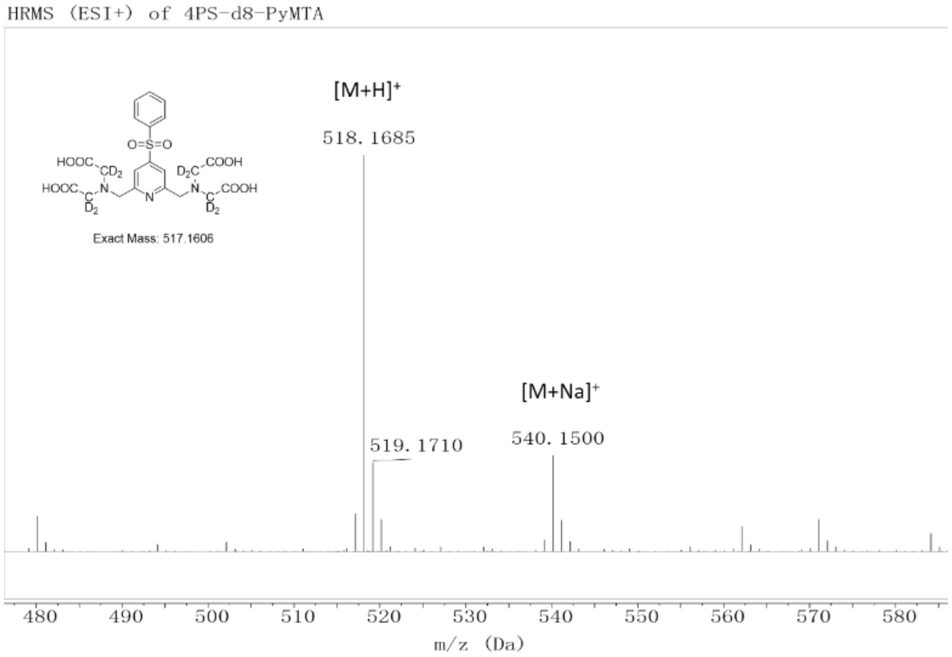

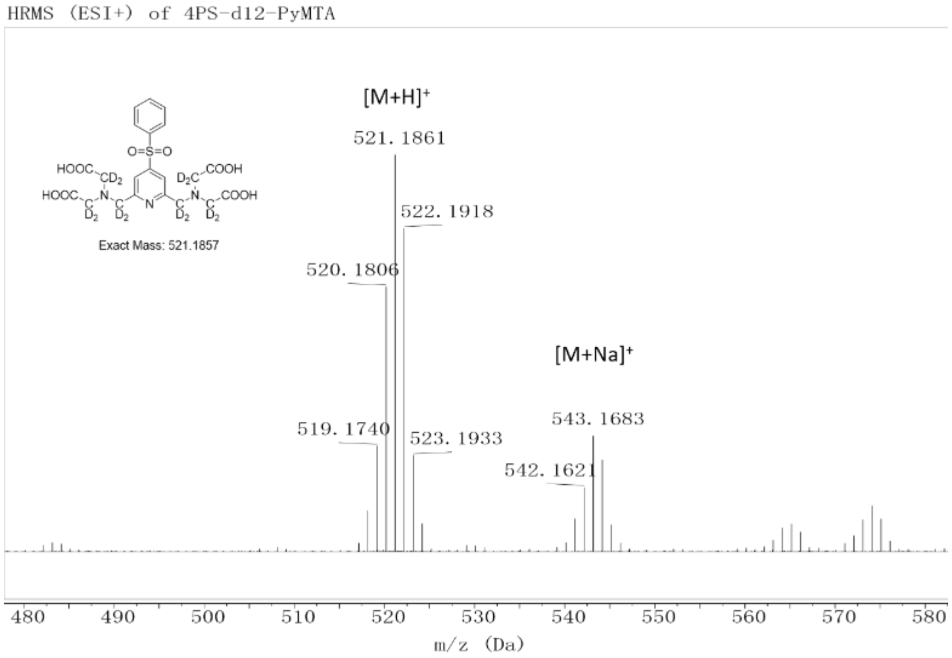

Figure S2. *High-resolution mass spectra of* ***4PS-d$_8$-PyMTA*** *and* ***4PS-d$_{12}$-PyMTA.*** *The spectra were recorded on an ESI-Q-TOF mass spectrometer.*

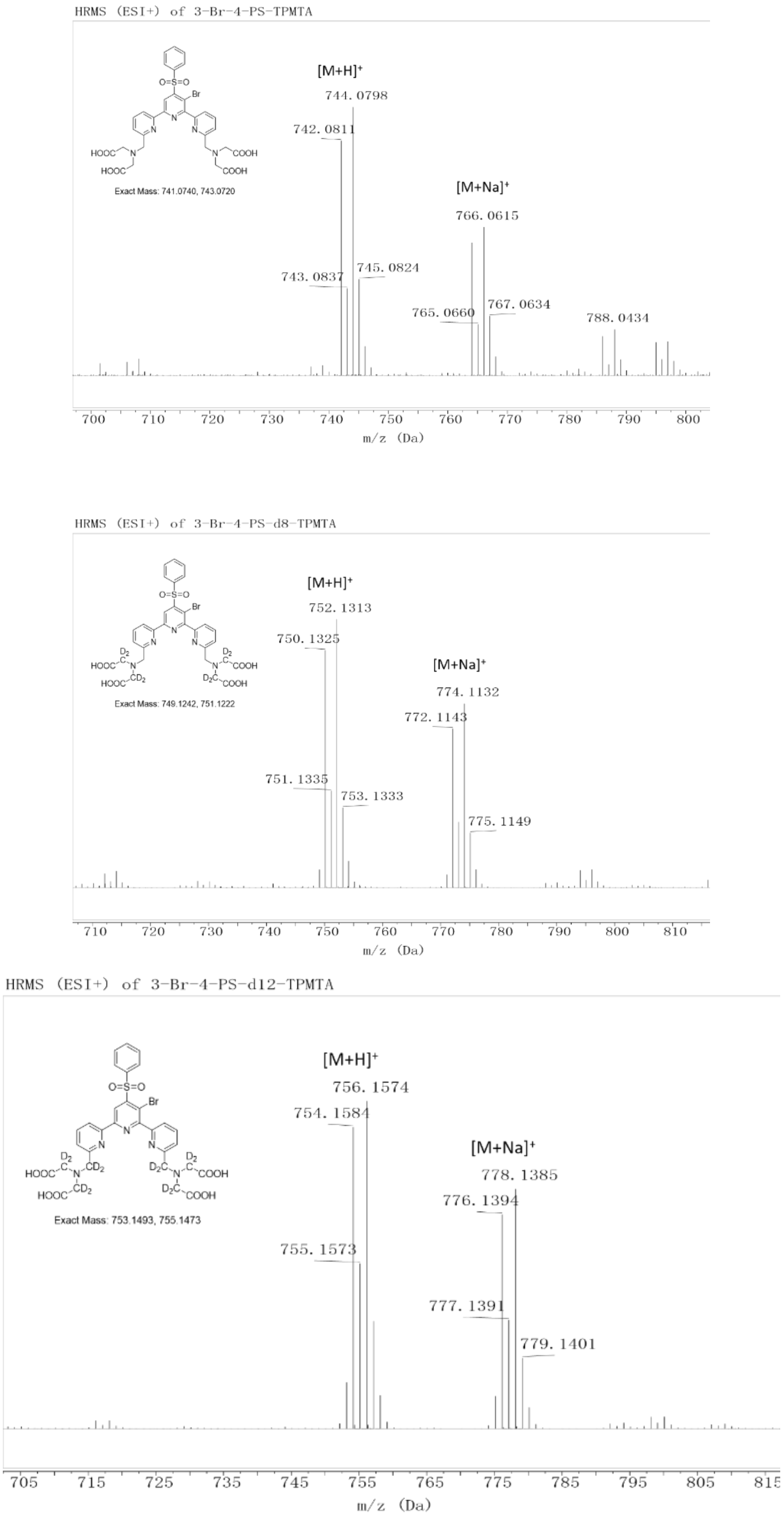

Figure S3. *High-resolution mass spectra of* ***3-Br-4PS-TPMTA, 3-Br-4PS-d_8-TPMTA*** *and* ***3-Br-4PS-d_{12}-TPMTA.*** *The spectra were recorded on an ESI-Q-TOF mass spectrometer.*

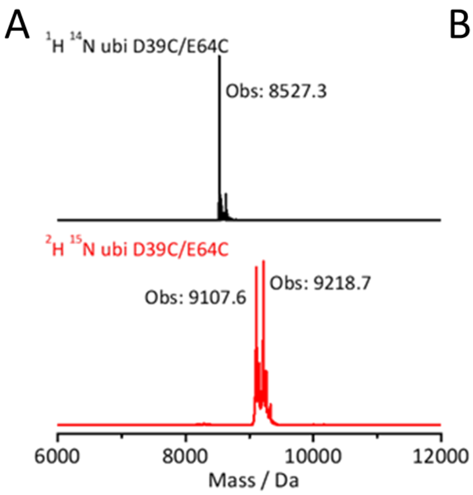

B

Protein	Predicted Mass	Observed Mass
^1H ^{14}N Ub D39C/E64C	8526.9	8527.3
^2H ^{15}N Ub D39C/E64C	9141.9	9107.6
Deuteration level in ^2H ^{15}N Ub D39C/E64C	94.4 % (assume 90% ^{15}N labeling efficiency)	

Figure S4. *(A) Mass spectra of purified ^1H, ^{14}N* ubiquitin *D39C/E64C and ^2H, ^{15}N* ubiquitin *D39C/E64C). (B) Isotopic labeling efficiencies. The spectra were recorded on an ESI-Q-TOF mass spectrometer. All exchangeable protons, such as -NH_2, -NH, -SH, -COOH, -OH, are calculated as ^1H. The molecular weight of 9218.7 observed for ^2H, ^{15}N* ubiquitin *D39C/E64C mass spectrum corresponds to the mass of ^2H, ^{15}N* ubiquitin *D39C/E64C in which a few exchangeable protons (^2H) are replaced by ^1H.*

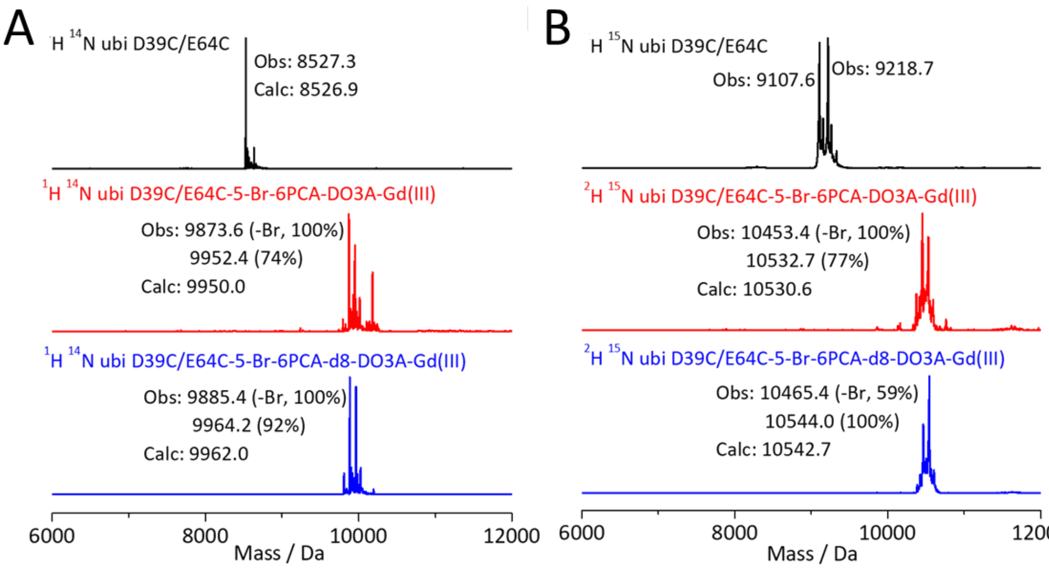

Figure S5. *The ESI-Q-TOF mass of* ubiquitin *D39C/E64C before and after **4PS-5-Br-6PCA-(d_n)-DO3A-Gd(III)** modification. (A) ^1H, ^{14}N* ubiquitin *D39C/E64C, (B) ^2H, ^{15}N* ubiquitin *D39C/E64C.*

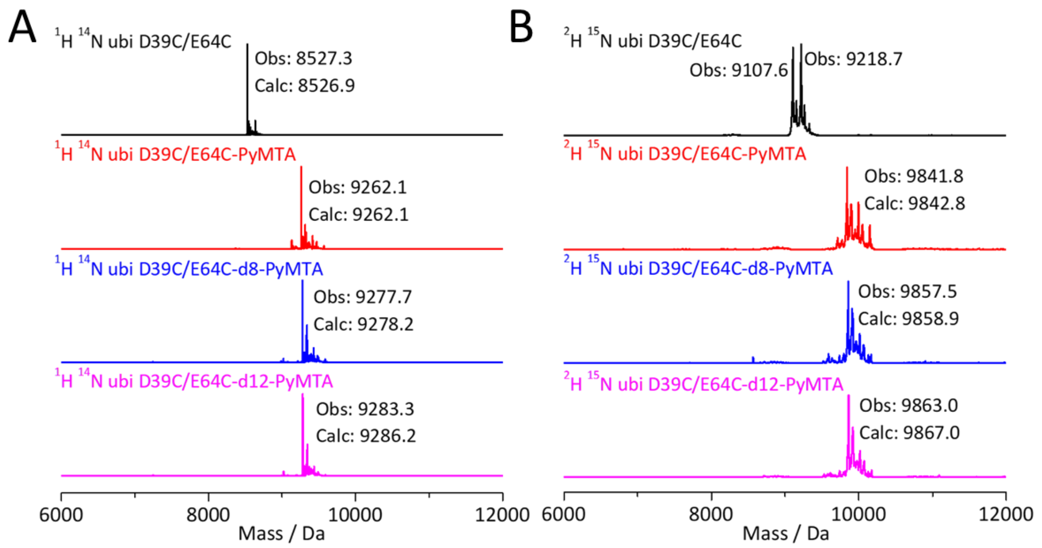

Figure S6. *The ESI-Q-TOF mass of ubiquitin D39C/E64C before and after* ***4PS-(d$_n$)-PyMTA*** *modification. (A)* ^{1}H, ^{14}N ubiquitin *D39C/E64C, (B)* ^{2}H, ^{15}N ubiquitin *D39C/E64C.*

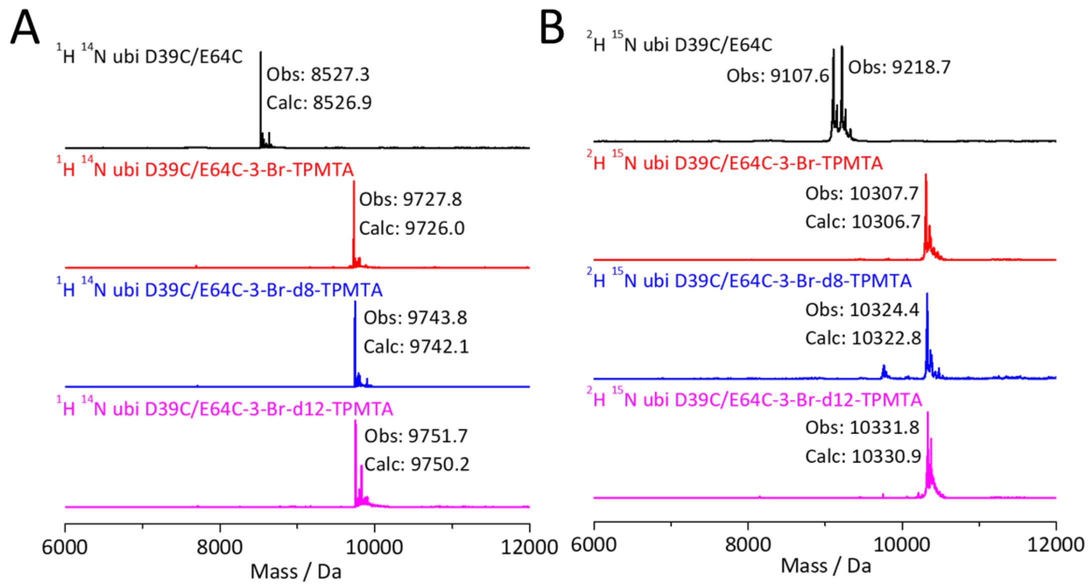

Figure S7. *The ESI-Q-TOF mass of ubiquitin D39C/E64C before and after* ***3-Br-4PS-(d$_n$)-TPMTA*** *modification. (A)* ^{1}H, ^{14}N ubiquitin *D39C/E64C, (B)* ^{2}H, ^{15}N ubiquitin *D39C/E64C.*

3. Mims ENDOR data

Table S1. *Hyperfine couplings of the various protons in Gd-PyMTA and Gd-TPMTA obtained from Mims ENDOR spectra and the corresponding Gd-H distances r.*

	Gd-PyMTA-d_n				Gd-TPMTA-d_n			
Hyperfine coupling $a_\perp$, kHz	1260-1720	900-920	440-450	140-170	1530-1940	940-990	450-510	200-270
Gd-H distance r^a, Å	3.5-3.9	4.3	5.5	7.6-8	3.4-3.7	4.2-4.3	5.5	6.5-7.2

[a] *r was calculated using the equation:* $a_\perp = \frac{\mu_0}{4\pi h}\left(\frac{g_e g_n \mu_e \mu_n}{r^3}\right)$, *where* μ_0 *is vacuum magnetic permeability,* g_e *and* g_n *are electron and nuclear g-values,* μ_B *and* μ_n *are Bohr magneton and nuclear magneton, respectively, h is the Planck constant, and r is the Gd–H distance; the hyperfine coupling was assumed to be purely dipolar.*

4. Examples of Hahn echo decay data

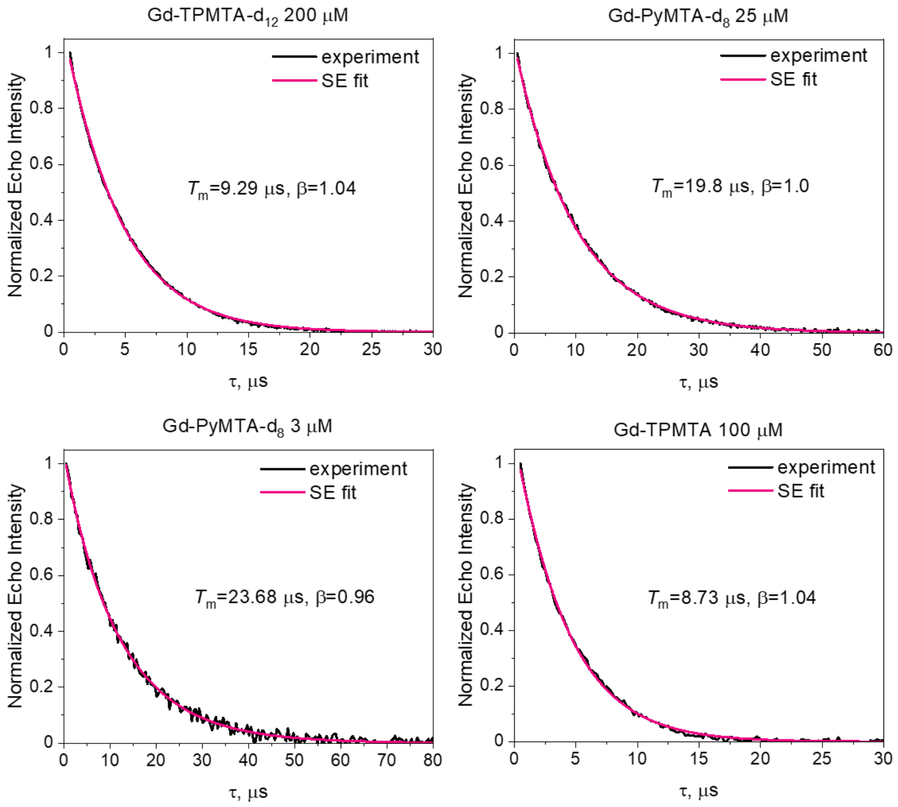

Figure S8. *Examples of Hahn echo decay data of samples that are indicated in the figure, and their fit to a stretched exponential function, the parameters of which are given in each panel.*

5. Testing for instantaneous diffusion

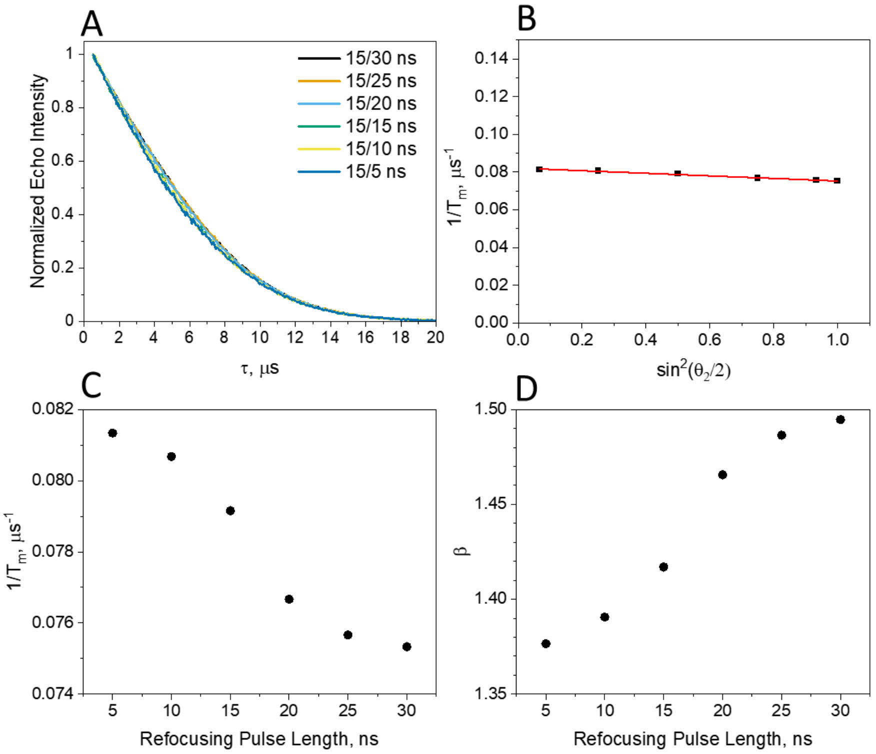

Figure S9. *(A) Normalized Hahn-echo decay traces of 200 μM Gd-PyMTA measured at the CT for a $\pi/2$ pulse of 15 ns and varying the second pulse (refocusing) lengths for a fixed power. (B) The dependence of $1/T_m$ on $\sin^2(\theta_2/2)$, where $1/T_m$ is given in (C) and θ_2 is the flip angle, with 30 ns corresponding to a π pulse (see eq. S1). Linear regression gave a slope of $-6.9\text{x}10^{-3}\pm4.2\text{x}10^{-4}$ μs^{-1} and an intercept of 0.082 ± 0.0002 μs^{-1}. The slope obtained shows a remarkable agreement with the calculated contribution for 200 μM according to eq. S1, giving a slope of $-6.94\text{x}10^{-3}$ μs^{-1}. This confirms the very small contribution of instantaneous diffusion to the phase relaxation. (C) The dependence of $1/T_m$, determined from fitting the traces in A, on the second pulse length. (D) The same as C for β.*

6. Inversion recovery data

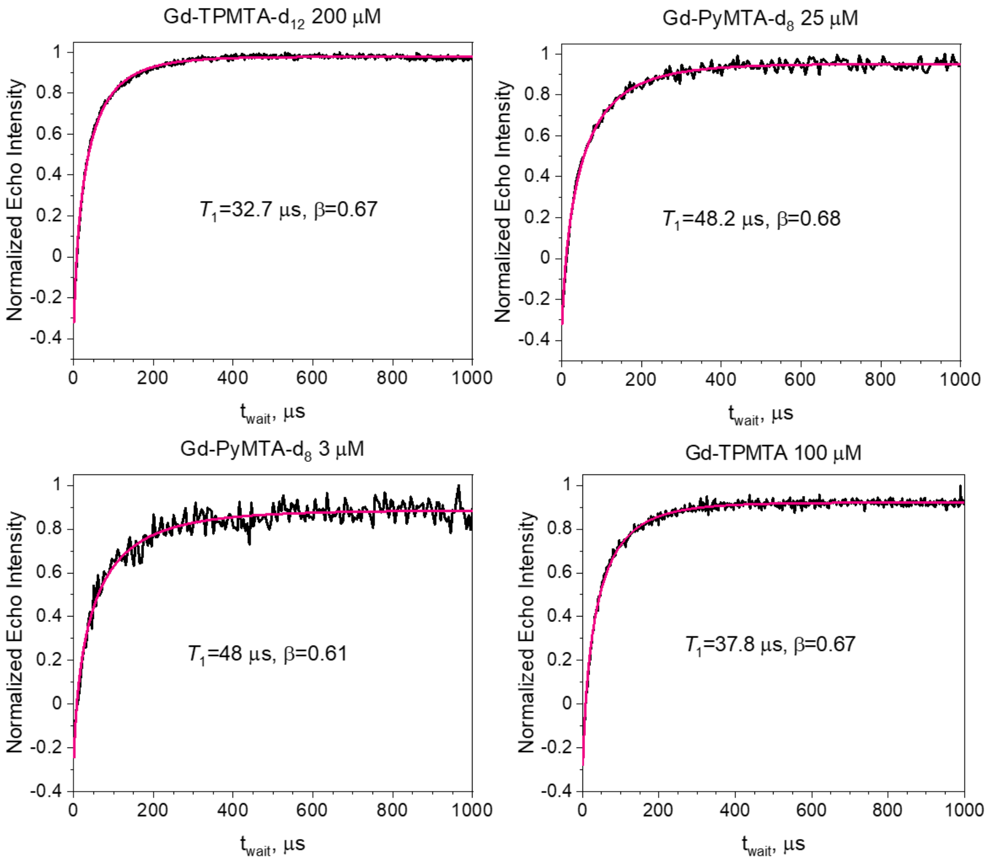

Figure S10. *Examples of inversion recovery data of the samples indicated in the figure, and the fitting parameters are noted in each panel.*

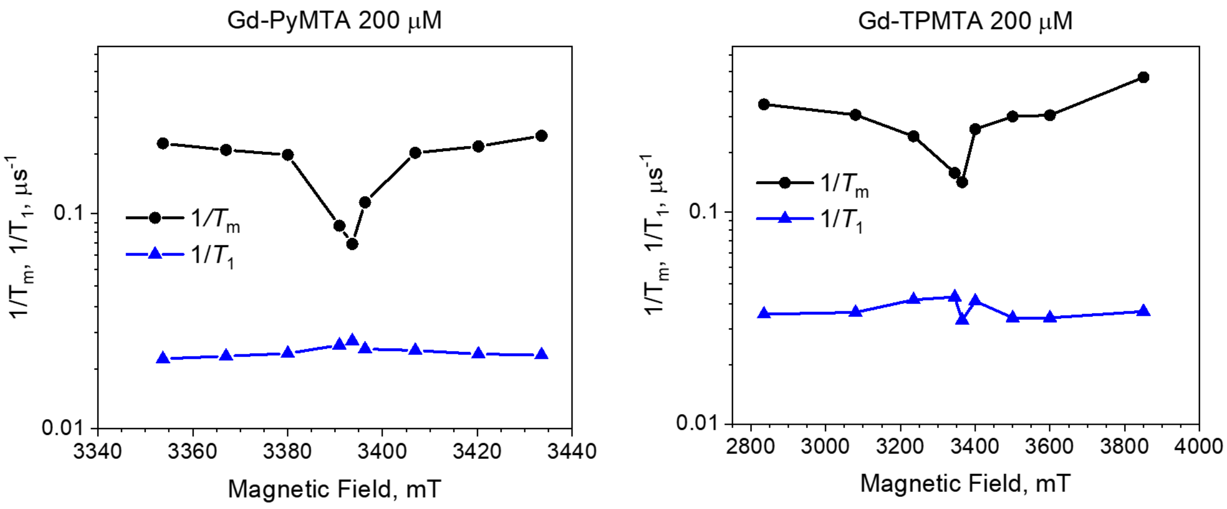

Figure S11. *Field dependence of $1/T_1$ and $1/T_m$ of the samples noted in the figure, measured at 10 K.*

7. Calculations of T_{ID} and T_{SD}

The contribution of T_{ID} to the echo decay is given by: (K. M. Salikhov, 1979; Mitrikas, 2023)

$$\frac{1}{T_{ID}} = \frac{(2S_A+1)}{4}\frac{8\pi^2}{9\sqrt{3}}\frac{\mu_0}{4\pi\hbar}g_A^2\mu_B^2 C_A sin^2\frac{\theta_2}{2} \quad \text{(S1)}$$

where C_A is the concentration of the A spins (spins per volume), and θ_2 is the rotation angle of the second pulse in the Hahn echo sequence. We calculated $1/T_{ID}$ as a function of concentration for Gd-PyMTA and Gd-TPMTA, assuming that *θ_2=180°.* This is an approximation, as it should be averaged over the hole burned in the EPR spectrum for broad-line EPR spectra. We estimated the A spins concentration to be 7% and 2% of the total spin population (based on DEER modulation depth measurements) for Gd-PyMTA and Gd-TPMTA, respectively. The results of the calculations are given in **Fig. S12**. The contribution of $1/T_{ID}$ is negligible, in agreement with the experimental results. We also calculated the $T_{SD,T1}$ for the two complexes according to : (K. M. Salikhov, 1979; Mitrikas, 2023)

$$\frac{1}{T_{SD,T1}} = \frac{1}{1.4}\sqrt{\frac{(2S_B+1)}{4}\frac{8\pi^2}{9\sqrt{3}}\frac{\mu_0}{4\pi\hbar}g_A\, g_B\mu_B^2 C_B/T_1^B} \quad \text{(S2)}$$

and the results are also shown in **Fig. S12**, taking C_B~C. The contribution of $1/T_{SD,T1}$ is significant. Comparison with **Fig. 4a** and **d** shows that it is somewhat overestimated compared to the experimental results. Considering that T_1 is underestimated as it was measured by inversion recovery, polarization was not considered, and the stretch exponential character of the measured $1/T_m$, the agreement is reasonable. Unfortunately, the spread of the experimental points cannot reliably reproduce the shape at the low concentration end.

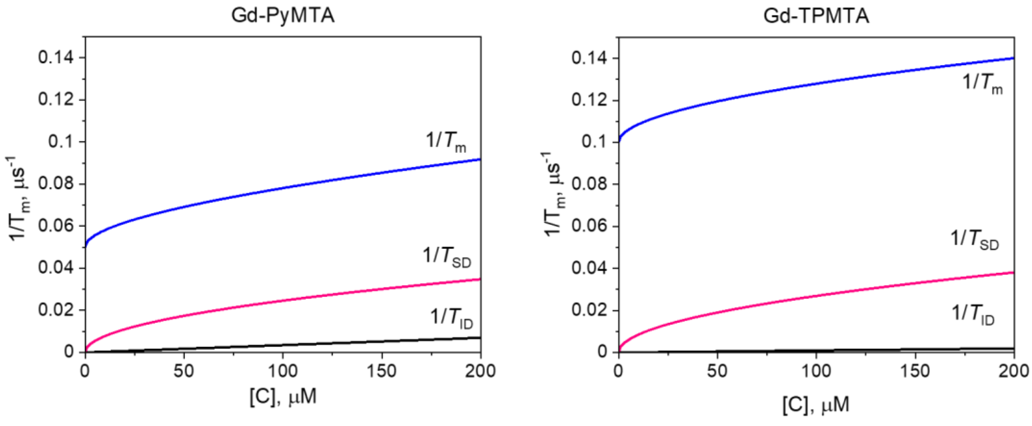

Figure S12. *Calculated concentration dependence of $1/T_{ID}$ (black) and $1/T_{SD}$ (red) and their sum (red) together with the experimental $1/T_m(0)$ for Gd-PyMTA (T_1=42 μs) and Gd-TPMTA (T_1=35 μs).*

8. Examples of 2-5 CP echo decays.

Table S2: The phase cycles used for the CP1-5 experiments, where "+" = "+*x*", "–" = "–*x*". Phase cycling was performed on every π-pulse, except for the last one, to eliminate the unwanted echo pathways. The $\pi/2$ pulse phase cycle was applied to compensate for baseline effects. The phase of the $\pi/2$ pulse always corresponded to the phase of the detector

CP1

#	π/2	π	detect
1	+	+	+
2	–	+	–

CP2

#	π/2	π_1	π_2	detect
1	+	+	+	+
2	+	–	+	+
3	–	+	+	–
4	–	–	+	–

CP3

#	π/2	π_1	π_2	π_3	detect
1	+	+	+	+	+
2	+	+	–	+	+
3	+	–	+	+	+
4	+	–	–	+	+
5	–	+	+	+	–
6	–	+	–	+	–
7	–	–	+	+	–
8	–	–	–	+	–

CP4

#	π/2	π_1	π_2	π_3	π_4	detect
1	+	+	+	+	+	+
2	+	+	+	–	+	+
3	+	+	–	+	+	+
4	+	+	–	–	+	+
5	+	–	+	+	+	+
6	+	–	+	–	+	+
7	+	–	–	+	+	+
8	+	–	–	–	+	+
9	–	+	+	+	+	–
10	–	+	+	–	+	–
11	–	+	–	+	+	–
12	–	+	–	–	+	–

13	−	−	+	+	+	−
14	−	−	+	−	+	−
15	−	−	−	+	+	−
16	−	−	−	−	+	−

CP5

#	π/2	π_1	π_2	π_3	π_4	π_5	detect
1	+	+	+	+	+	+	+
2	+	+	+	+	−	+	+
3	+	+	+	−	+	+	+
4	+	+	+	−	−	+	+
5	+	+	−	+	+	+	+
6	+	+	−	+	−	+	+
7	+	+	−	−	+	+	+
8	+	+	−	−	−	+	+
9	+	−	+	+	+	+	+
10	+	−	+	+	−	+	+
11	+	−	+	−	+	+	+
12	+	−	+	−	−	+	+
13	+	−	−	+	+	+	+
14	+	−	−	+	−	+	+
15	+	−	−	−	+	+	+
16	+	−	−	−	−	+	+
17	−	+	+	+	+	+	−
18	−	+	+	+	−	+	−
19	−	+	+	−	+	+	−
20	−	+	+	−	−	+	−
21	−	+	−	+	+	+	−
22	−	+	−	+	−	+	−
23	−	+	−	−	+	+	−
24	−	+	−	−	−	+	−
25	−	−	+	+	+	+	−
26	−	−	+	+	−	+	−
27	−	−	+	−	+	+	−
28	−	−	+	−	−	+	−
29	−	−	−	+	+	+	−
30	−	−	−	+	−	+	−
31	−	−	−	−	+	+	−
32	−	−	−	−	−	+	−

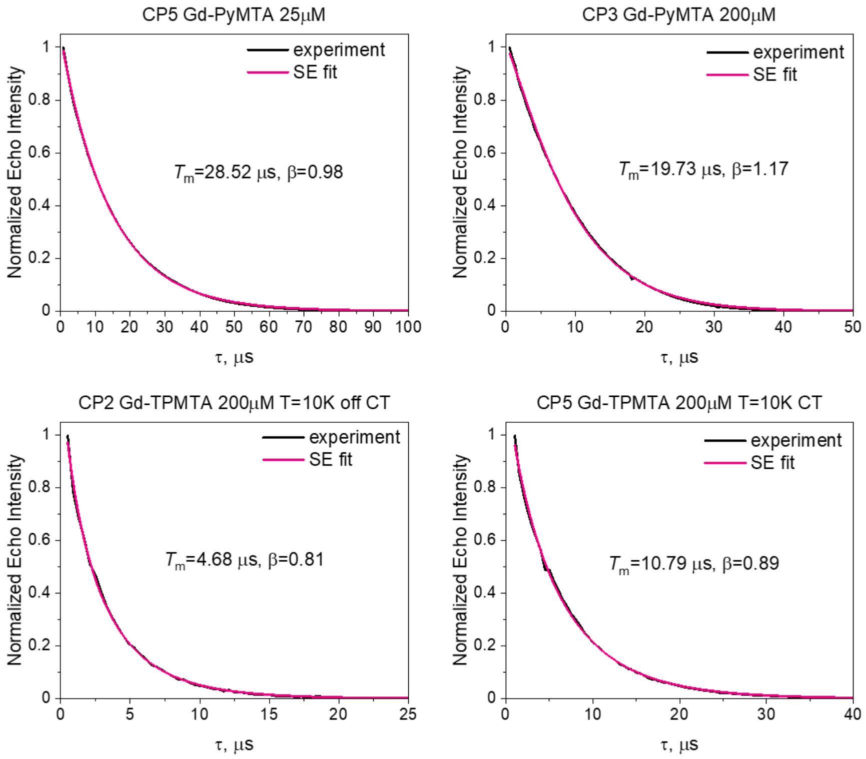

***Figure S13**. Examples of echo decays for CP experiments with n=2-5 for the samples noted in the figure and their fit to a stretched exponential function. The fitting parameters are given in each panel.*

9. Effect of stimulated echo on full CP train decay

To evaluate the effect of overlapping stimulated echoes on the refocused echoes in the CP trains, we carried out calculations of the echo train taking into account the pulses' bandwidth (duration) along with the EPR linewidth according to earlier reports (Zaripov et al., 2021; Mitrikas, 2023). We assumed an effective spin of S=1/2 and focused on Gd-PyMTA, which has a narrow CT with a FWHH (full width at half height) of 60 MHz. In **Fig. S14**, we present the stimulated and refocused echo produced for *n*=2 and $\tau_1 \neq \tau_2$, along with simulation results. We see that the stimulated echo intensity is significant, and the shape and relative intensity of the two echoes can be reproduced rather well.

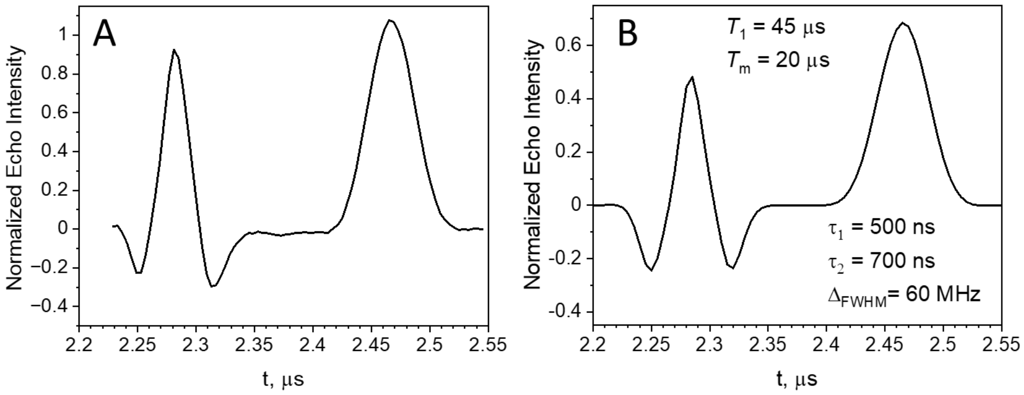

Figure S14. *(A) The experimentally observed stimulated (first) and the refocused echo (second), obtained with n=2 and $\pi/2$ and π of 15 and 30 ns and τ_1=500 ns and τ_2=700 ns. Measurements were at the maximum of the CT of Gd-PyMTA. (B) Simulations with the pulse lengths and pulse intervals used in the experiment, an EPR linewidth of 60 MHz, and the T_1 and T_m values are noted in the figure.*

Next, we calculated the full CP train with the sequence parameters used in the experiment. Simulations were carried out for a spectral width of 60 MHz (selective excitation) and for 6 MHz (non-selective excitation) for T_m=20 μs and T_1=45 μs, parameters like those found in our samples. The echo train decays in the two cases, given in **Fig. S15**, are very similar and could be simulated with an exponential decay with β=1 and very similar T_m values of 20.120 and 20.095 μs for the selective and non-selective pulses, respectively. This analysis was done with integrated echos. We note that the width of the integration window affects the derived T_m values because of the shape of the stimulated echo (see Fig. **S14**). This result is quite surprising because the formation of the stimulated echoes for the selective pulses should introduce contributions of T_1. A possible reason that the T_m's for selective and non-selective pulses are so close could be that the values of T_m and T_1 are close. In the ideal case where T_m=T_1, one should not see a difference.

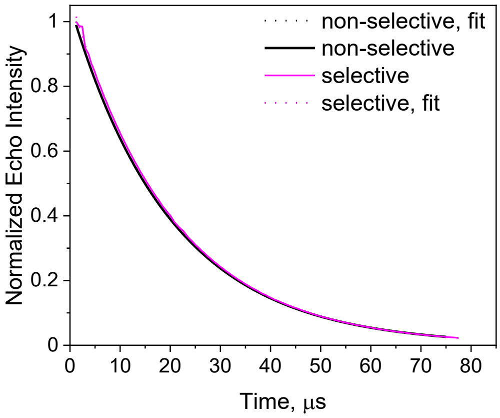

Figure S15. *The full CP train decays, calculated for non-selective and selective pulses (same parameters as in **Fig. S14**) with T_m=20 μs, T_1=45 μs, τ=290 ns, and the corresponding fits.*

To explore the effect of T_1 we carried out simulations of the full CP train for T_m=15 μs, τ=290 ns and T_1=45 μs or T_1=45 ms for a spectral width of 60 MHz (selective pulses). The results are given in **Fig. S16**. **Fig. S16A** compares the experimental train with the two calculated traces; zooms on the early and late times are presented in **Figs. S16 B,C**. The decay traces, presented in **Fig. 16 D,E**, are very similar and could be simulated with a single stretched exponential decay with T_m=13 μs and β=0.89 for T_1=45 μs and T_m=13.3 μs and β=0.88 for T_1=45 ms. This indicates that the effect of the stimulated echo is minor and causes underestimation of T_m once the echos' intensity are determined by integration. As shown by Mitrikas and co-workers, the effect of selective pulses is twofold: on one hand, they enhance the decay of the refocused echo due to the redistribution of the echo profile as a result of selective excitation and on the other hand, they slow its decay by overlapping with stimulated echoes (an extensive theoretical and experimental study of the effect of selective pulses on CPMG decays will be published elsewhere by the Mitrikas lab). These contrasting effects may result in only a modest change in T_m, as we observed. The presented simulations show that the two distinct populations with different relaxation properties, observed in the Gd(III) full CP train experiments, are not a consequence of the unwanted stimulated echoes due to selective pulses.

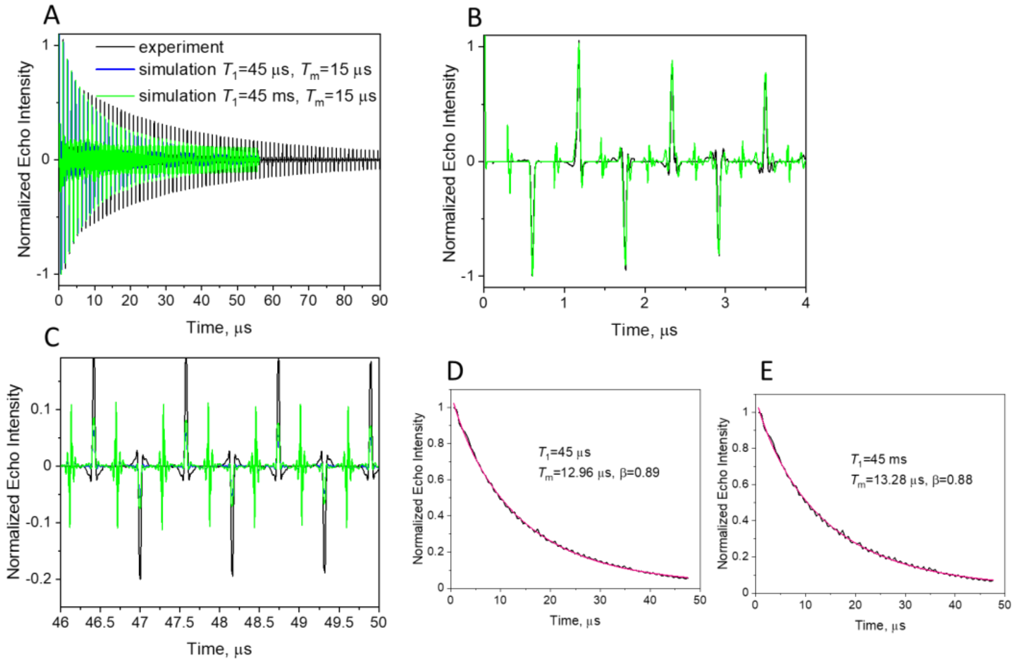

Figure S16. *(A) Experimental (black) full CP train of Gd-PyMTA (25 μM, 10 K), recorded with π/2 and π pulses of 15 and 30 ns, τ=290 ns, with simulations using these parameters and EPR linewidth of 60 MHz, T_m=15 μs and T_1=45 μs (blue) and T_1=45 ms (green). (B,C) Same as (A) zooming at different time regions. (D,E) Decays of the calculated echo trains and their fits.*

Finally, to ensure that the observation of two populations in the Gd(III) full CP train decays is not due to an experimental artifact, we carried out similar measurements on a nitroxide spin label, 100 μM MTSL dissolved in D_2O/glycerol-d_8 (25% glycerol v/v), shown in **Fig. S17**. The measurements were carried out at 40 K at the maximum of the ED-EPR spectrum (**Fig. S17A)** with π/2 and π pulse lengths of 20 and 40 ns. The Hahn echo decay could be fitted with a

single stretched exponent (Fig. **S17B**), and the values are given in the figure. The T_1 measurements are shown in **Fig. S17C**. The spectrum of the nitroxide is broader than the CT of Gd-PyMTA and the pulses are longer; therefore, the stimulated echo's intensity is larger than the refocused echo for *n*=2 (**Fig. S17D**). The CP train is presented in **Fig. S17E**, and its decay, given in **Fig. S17F,** could be fitted with a single stretched exponent with parameters depicted in the figure. This further supports that the observation of two populations for the Gd-complexes is intrinsic to the Gd(III) samples and is not an experimental artifact. Interestingly, Q-band relaxation data published recently on nitroxide radicals reported that Hahn echo decay had to be fitted with two populations, using the so-called SSE mechanism (Soetbeer et al., 2021), in contrast to one population observed at W-band.

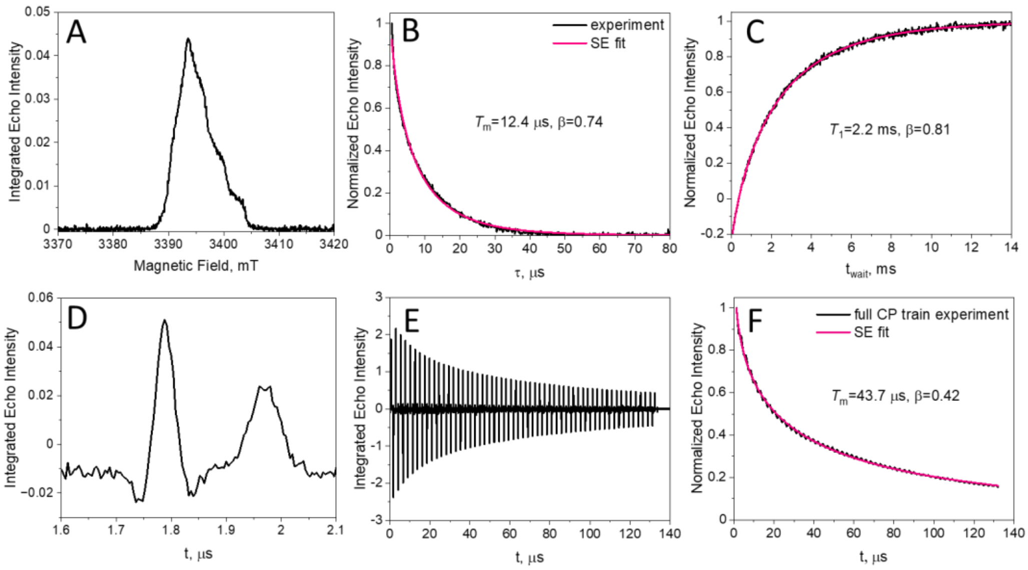

Figure S17. *(A) ED-EPR of 100 μM MTSL dissolved in D_2O/glycerol-d_8 (25% glycerol v/v) recorded at 40 K. (B) Hahn echo decay measured at the maximum of the ED-EPR spectrum and the fit with one stretched exponential decay. (C) Inversion recovery experiment to determine T_1 and β. (D) The echoes observed for CP2 and intervals of τ_1=500 ns and τ_2=700 ns; the first is the stimulated echo, and the second is the refocused echo. (E) The full CP train. (F) The decay of the echoes in the train and its fit. All fit parameters are shown in the corresponding panels.*

10. Example of Hahn echo decays and CP data on labeled proteins.

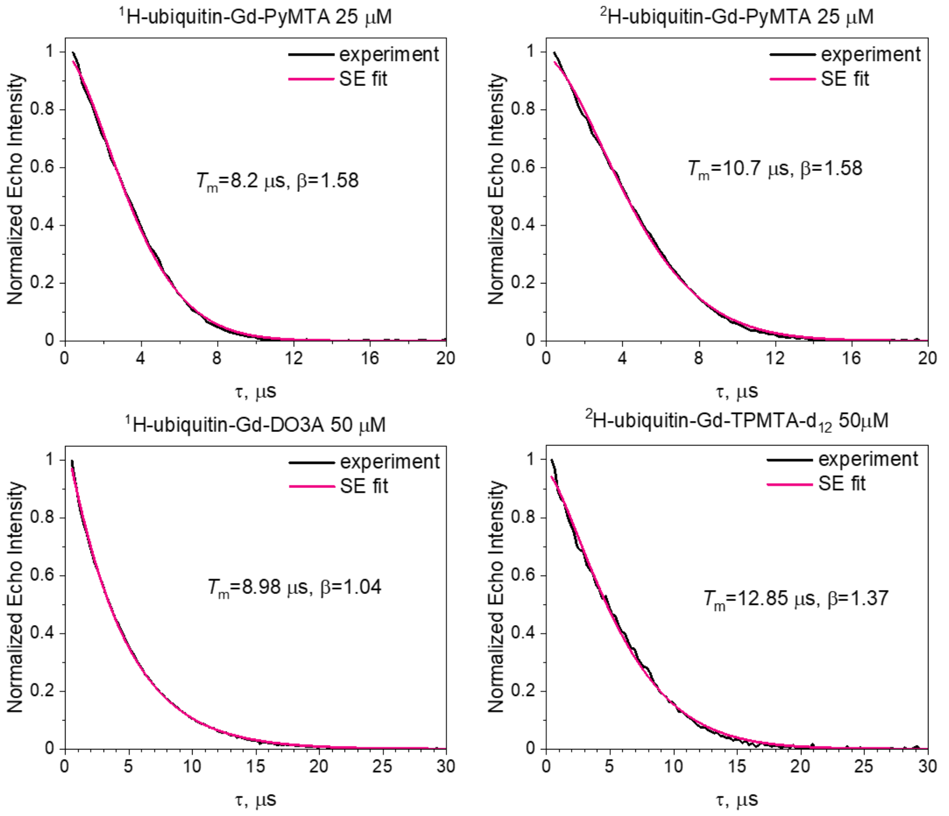

Figure S18. *Examples of echo decays of the spin-labeled proteins noted in the figure, and their fit to a stretched exponential function. The fitting parameters are given in each panel.*

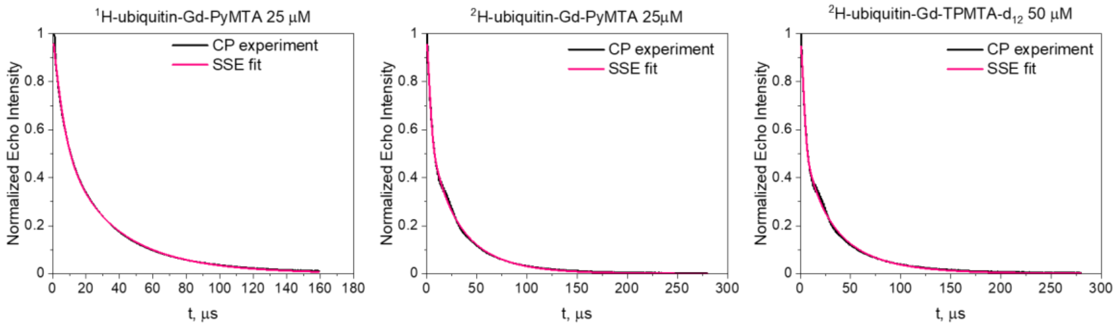

Figure S19. *Examples of full CP train decay of the spin-labeled proteins noted in the figure and their fit to a sum of two stretched exponential functions with the following parameters:* $T_{m,f}$=6.41 μs, β_f=1.24, A=0.28, $T_{m,s}$=26.99 μs, β_s=0.86 *for* ^{1}H-ubi Gd-PyMTA; $T_{m,f}$=7.43 μs, β_f=1.04, A=0.19, $T_{m,s}$=26.22 μs, β_s=0.79 *for* ^{2}H-ubi Gd-PyMTA; *and* $T_{m,f}$=5.37 μs, β_f=2.46, A=0.34, $T_{m,s}$=20.22 μs, β_s=0.77 *for* ^{2}H-ubi Gd-TPMTA-d_{12}.

11. Effect on HEPES and glycerol content of the Hahn echo decay of Gd-PyMTA

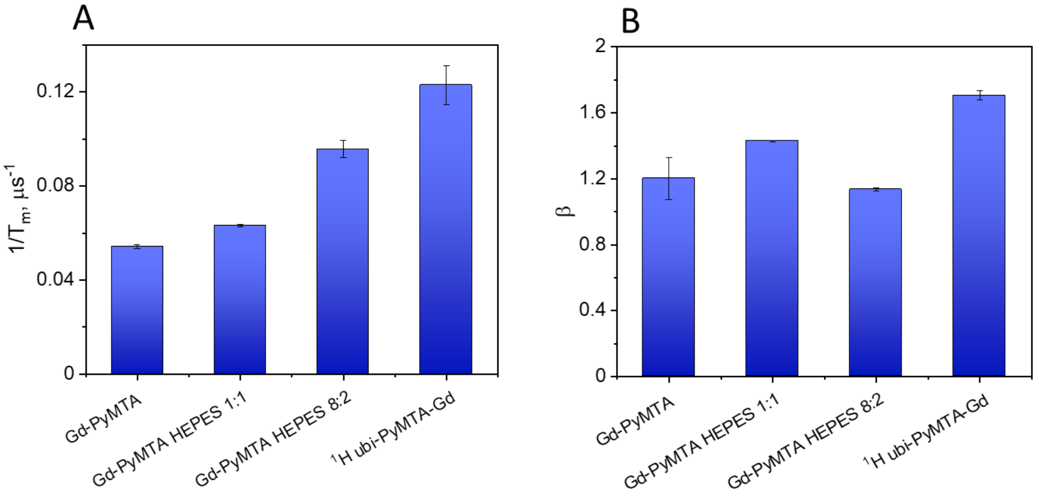

Figure S20. *Comparison of $1/T_m$ (A) and β (B) for Hahn echo decays for different complexes with concentrations [C]=25 µM for Gd-PyMTA and ubiquitin labeled with Gd-PyMTA. The ratios refer to the D_2O:glycerol-d_8 in the sample (v/v) and HEPES refers to the presence of the HEPES buffer.*

12. DEER data.

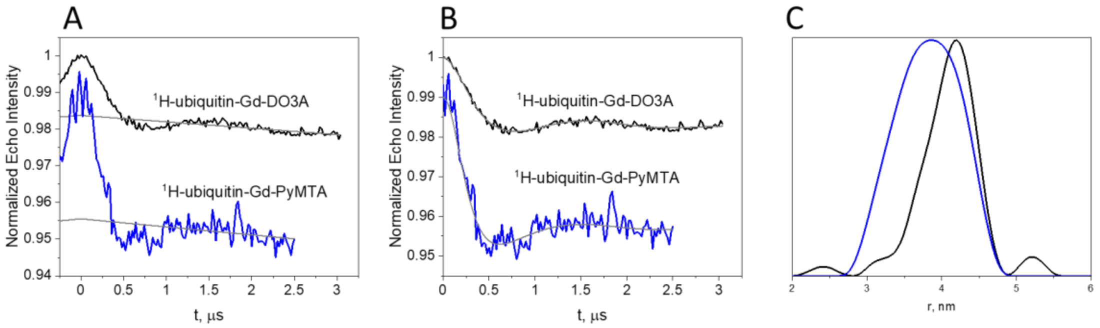

Figure S21. *DEER data of ubiquitin D39C/E64C labeled with Gd-DO3A and Gd-PyMTA. (A) The primary normalized DEER traces with the background decay function. (B). The DEER traces after background subtraction with the fitted data obtained with Tikhonov regularization using DEER analysis (Jeschke et al., 2006). (C) The corresponding distance distribution.*

13. Table with overview of T_m and β values

Table S3 : Overview of the T_m and β values of the samples studied in this work, measured by Hahn echo at the CT and 10 K.

Sample	Conc. (μM)	T_m (μs)		β	
Gd-PyMTA	50	16.53 ± 0.77		1.21 ± 0.07	
Gd-PyMTA-d$_8$,	50	19.59 ± 0.31		1.14 ± 0.05	
Gd-PyMTA-d$_{12}$	50	18.41 ± 0.84		1.16 ± 0.08	
Gd-TPMTA[a]	50	9.96 ± 0.57		0.92 ± 0.03	
Gd-TPMTA-d$_8$[a]	50	10.09 ± 1.33		0.94 ± 0.07	
Gd-TPMTA-d$_{12}$[a]	50	9.75 ± 0.42		0.90 ± 0.04	
		^{1}H protein	^{2}H protein	^{1}H protein	^{2}H protein
Ubi-Gd-PyMTA	25	8.1 ± 0.04	9.73 ± 0.05	1.43 ± 0.01	1.38 ± 0.01
Ubi-Gd-PyMTA-d$_8$	25	8.25 ± 0.04	9.1 ± 0.05	1.33 ± 0.01	1.27 ± 0.01
Ubi-PyMTA-d$_{12}$	25	8.18 ± 0.03	9.1 ± 0.07	1.35 ± 0.01	0.99 ± 0.01
Ubi-Gd-TPMTA[a]	50	5.4 ± 0.6	5.2 ± 0.3	1.19 ± 0.04	0.99 ± 0.01
Ubi-Gd-TPMTA-d$_8$[a]	50	6.2 ± 0.6	6.6 ± 0.4	1.02 ± 0.05	1.00 ± 0.02
Ubi-Gd-TPMTA-d$_{12}$[a]	50	5.6 ± 0.3	9.1 ± 0.05	1.02 ± 0.02	1.27 ± 0.01
Ub-Gd-DO3A	50	8.98 ± 0.04	8.09 ± 0.04	1.05 ± 0.01	0.912 ± 0.004
Ub-Gd-DO3A-d$_8$	50	8.77 ± 0.04	9.34± 0.05	1.014 ± 0.004	0.916 ± 0.004

[a]Average of measurements carried out at fields 1,2, and 3.

References

Jeschke, G., Chechik, V., Ionita, P., Godt, A., Zimmermann, H., Banham, J., Timmel, C. R., Hilger, D., and Jung, H.: DeerAnalysis2006—a comprehensive software package for analyzing pulsed ELDOR data, Appl. Magn. Reson., 30, 473-498, 10.1007/BF03166213, 2006.

K. M. Salikhov, Y. D. T.: Electron spin-echo studies of spin-spin interactions in solids, in: Time Domain Electron Spin Resonance, edited by: Kevan, L. S., Robert N., Wiley, NY, 231-298, 1979.

Kadjane, P., Platas-Iglesias, C., Ziessel, R., and Charbonnière, L. J.: Luminescence properties of heterodinuclear Pt–Eu complexes from unusual nonadentate ligands, Dalton Trans., 5688-5700, 10.1039/B903522B, 2009.

Li, C. and Wong, W.-T.: A selective one-step synthesis of tris N-alkylated cyclens, Tetrahedron, 60, 5595-5601, https://doi.org/10.1016/j.tet.2004.04.086, 2004.

Li, J. and Byrd, R. A.: A simple protocol for the production of highly deuterated proteins for biophysical studies, J. Biol. Chem., 298, 102253, https://doi.org/10.1016/j.jbc.2022.102253, 2022.

Mitrikas, G.: Long Electron Spin Coherence Times of Atomic Hydrogen Trapped in Silsesquioxane Cages, J. Phys. Chem. Lett., 14, 9590-9595, 10.1021/acs.jpclett.3c02626, 2023.

Montgomery, J. R. D., Lancefield, C. S., Miles-Barrett, D. M., Ackermann, K., Bode, B. E., Westwood, N. J., and Lebl, T.: Fractionation and DOSY NMR as Analytical Tools: From Model Polymers to a Technical Lignin, ACS Omega, 2, 8466-8474, 10.1021/acsomega.7b01287, 2017.

Soetbeer, J., Millen, M., Zouboulis, K., Hülsmann, M., Godt, A., Polyhach, Y., and Jeschke, G.: Dynamical decoupling in water–glycerol glasses: a comparison of nitroxides, trityl radicals and gadolinium complexes, Phys.Chem.Chem.Phys., 23, 5352-5369, 10.1039/D1CP00055A, 2021.

Wang, G., Liang, X., Chen, L., Gao, Q., Wang, J.-G., Zhang, P., Peng, Q., and Xu, S.: Iridium-Catalyzed Distal Hydroboration of Aliphatic Internal Alkenes, Angew. Chem. Intl. Ed., 58, 8187-8191, https://doi.org/10.1002/anie.201902464, 2019.

Yang, Y., Wang, J.-T., Pei, Y.-Y., and Su, X.-C.: Site-specific tagging proteins via a rigid, stable and short thiolether tether for paramagnetic spectroscopic analysis, Chem. Comm., 51, 2824-2827, 10.1039/C4CC08493D, 2015.

Yang, Y., Yang, F., Li, X.-Y., Su, X.-C., and Goldfarb, D.: In-Cell EPR Distance Measurements on Ubiquitin Labeled with a Rigid PyMTA-Gd(III) Tag, J. Phys.Chem.B, 123, 1050-1059, 10.1021/acs.jpcb.8b11442, 2019.

Zaripov, R., Khairutdinov, I., and Salikhov, K.: Specific Features of Studying the Paramagnetic Relaxation of Spins by the Carr–Purcell–Meiboom–Gill Method Related to the Superposition of Echo Signals, Russ.J.Phys.Chem.B, 15, 389-393, 10.1134/S1990793121030337, 2021.
